# Food Consumption Patterns of Infants and Toddlers: Findings from the Feeding Infants and Toddlers Study (FITS) 2016

**DOI:** 10.1093/jn/nxy171

**Published:** 2018-08-31

**Authors:** Amira A Roess, Emma F Jacquier, Diane J Catellier, Ryan Carvalho, Anne C Lutes, Andrea S Anater, William H Dietz

**Affiliations:** 1 The George Washington University, Milken Institute School of Public Health, Washington, DC; 2 Nestlé Research, Lausanne, Switzerland; 3 RTI International, Research Triangle Park, NC; 4 Nestlé Nutrition, Florham Park, NJ

**Keywords:** Feeding Infants and Toddlers Study, FITS 2016, food intakes, breastfeeding, eating habits, young children, disparities

## Abstract

**Background:**

The prevalence of obesity and type 2 diabetes continues to increase. These conditions disproportionately affect minorities and are associated with poor nutrition early in life. Current food-consumption patterns can inform pending dietary guidelines for infants and toddlers.

**Objective:**

The aim of this study was to describe infant feeding, complementary feeding, and food and beverage consumption patterns of 0- to 23.9-mo-olds in the general population.

**Methods:**

The Feeding Infants and Toddlers Study 2016 is a cross-sectional survey of caregivers of children aged <4 y. Dietary data were collected from a national random sample by using a 24-h dietary recall (*n *= 3235). The percentage of children consuming foods from >400 food groups was calculated. Differences in the percentage consuming between Hispanic, non-Hispanic white, and non-Hispanic black children aged 0–23.9 mo were evaluated with the use of ORs and 95% CIs.

**Results:**

Eighty-three percent of 0- to 23.9-mo-olds (*n *= 2635) were ever breastfed, 34% of 0- to 3.9-mo-olds (*n *= 305) and 15% of 4- to 5.9-mo-olds (*n *= 295) were exclusively breastfed, and 24% of 12- to 14.9-mo-olds (*n *= 412) consumed breast milk on the day of the recall. Complementary foods were more likely to be introduced before 4 mo in formula-fed infants (27%) than in infants who did not consume formula (5%). Half of 4- to 5.9-mo-olds consumed iron-fortified infant cereal, but few consumed iron-rich meats. Among toddlers (12–23.9 mo; *n* = 1133), >20% consumed no servings of fruit or vegetables on the day of the recall, approximately half consumed 100% fruit juice, and one-quarter to one-third consumed a sugar-sweetened beverage (SSB).

**Conclusions:**

Breastfeeding initiation and duration have improved, but exclusivity remains low. Low consumption of iron-rich foods, fruit, and vegetables and lack of variety in vegetable consumption are problems. Efforts to reduce the consumption of SSBs and 100% fruit juice are warranted in early childhood.

## Introduction

Approximately 8% of US infants <2 y old have high weight relative to recumbent length (1), and 14% of 2- to 5-y-olds are obese (2). Obesity at age 5 y is a strong predictor of obesity in early adolescence (3). Breastfeeding is associated with a lower risk of later development of overweight, obesity, and type 2 diabetes (4, 5); however, some studies have questioned the association between breastfeeding and lower risk of other health outcomes, such as cholesterol and blood pressure (4, 6). The relation between age of introduction of complementary foods, however, and later development of overweight and obesity is not consistent (5). The identification of the foods and dietary behaviors that may contribute to excess caloric

intake can offer insights into dietary strategies to prevent obesity and other poor health outcomes. In addition, previous studies have shown that low-income and some minority populations are disproportionately affected by these poor health outcomes (7–12). Consequently, an exploratory consideration of potential racial/ethnic differences in dietary intake may help to guide further research and policy discussions to support more vulnerable populations.

The American Academy of Pediatrics (AAP) has provided comprehensive recommendations for early feeding practices for children <2 y old. Optimal breastfeeding behaviors recommended by the AAP include exclusive breastfeeding for the first 6 mo of life and continued breastfeeding until ≥12 mo (9). Breastfeeding reduces the risks of sudden infant death syndrome, infectious diseases, asthma, obesity, and type 2 diabetes (13–18), all of which disproportionately affect minority children (12, 19–21). Model estimates of the excess disease cases, deaths, and costs attributable to suboptimal breastfeeding are substantial for both mothers and infants in the United States. For example, estimates are that optimal breastfeeding could prevent >700 child deaths in the United States annually (22). National breastfeeding rates remain low, particularly the rate of exclusive breastfeeding in the first 6 mo of life (23). Furthermore, low-income minority women, especially non-Hispanic black (NHB) mothers, breastfeed at lower rates than their non-Hispanic white (NHW) counterparts (24, 25).

The AAP Committee on Nutrition recommends the introduction of complementary foods between 4 and 6 mo of age (26, 27). At this age, breast milk alone is typically no longer sufficient to meet an infant's nutritional requirements without the introduction of other foods and liquids (complementary foods). The introduction of complementary foods represents a critical transition from the largely milk-based infant diet to a diet primarily based on the foods eaten by the rest of the family by the time the child is 18–24 mo old (28). The choice of complementary foods may affect intakes of essential nutrients that older infants and toddlers may underconsume, such as iron and vitamin D, as well as nutrients that are often consumed to excess, such as sodium. Small studies have reported that complementary feeding practices are more likely to depart from the AAP recommendations for minority children (29), but only limited nationally representative data are available to adequately quantify these differences (30).

Despite the links between early nutrition and health outcomes, national data on early feeding practices are largely absent from the literature. Furthermore, the Dietary Guidelines for Americans, first issued in 1980, have not yet covered children aged 0–24 mo (the so-called “Birth to 24” or “B-24” age group). However, the first B-24 recommendations are expected to be released in the 2020–2025 Dietary Guidelines for Americans (31).

The Feeding Infants and Toddlers Study (FITS) is a unique survey that provides national estimates of early feeding behaviors, including breastfeeding and introduction of complementary foods, which can inform the development of dietary guidelines for the B-24 age group. Previous FITS studies reported that the prevalence of children who were “ever breastfed” was 76% ± 1.1% (mean ± SE) in 2002 and modestly higher in 2008 (80% ± 1.5%). Those studies also report that infant cereal was introduced between 4 and 6 mo for only 65% of infants in 2002 and 50% in 2008 (32). The consumption of fruit and vegetables was substantially lower than recommendations in previous studies as well, with 15–30% of infants and toddlers >6 mo old consuming no fruit and 25–30% consuming no vegetables on the day of the survey in both the FITS 2002 and 2008 (32).

The aim herein is to provide a cross-sectional description of the breastfeeding practices, the use of human-milk substitutes, and complementary feeding behaviors among 0- to 23.9-mo-olds from the FITS 2016. In addition, exploratory analyses were performed to identify potentially important racial/ethnic differences. These results can inform the development of US dietary guidelines for children aged <2 y and lead to recommendations to optimize both breastfeeding and complementary feeding practices among the general population and, potentially, minorities. In addition, these findings can be used to indicate avenues for future research, such as the development of hypotheses to explore in the analysis of FITS 2002, 2008, and 2016 to compare trends over time.

## Methods

### FITS survey methods

A detailed report of the FITS 2016 design and methodology is reported elsewhere in this supplement issue (33). Briefly, the FITS 2016 is a national cross-sectional survey designed to collect data on the food and beverage intake, using the 24-h recall method, among children <4 y old living in the 50 states and Washington, DC. This method enables subsequent estimation of food and nutrient intakes on the day of the survey. Details of the questionnaire development and testing, sampling, and data collection methodology largely replicate the methods used in previous FITS surveys conducted in 2008 and 2002 (34, 35), with some updates to the food classification scheme. The questionnaires from 2008 were used in 2016 with minor modifications and the addition of some new data items (none of which are reflected in this article). The sample for FITS 2016 was identified through stratified random sampling from 4 sampling frames to obtain target sample sizes in cells defined by 12 age groups. In addition, a secondary objective of the target sample frames was to obtain adequate cell sizes within each of the 12 age groups of children participating in the Special Supplemental Nutrition Program for Women, Infants, and Children (WIC). Sampling weights were calculated to account for the probability of household selection and then adjusted for nonresponse and incomplete coverage by calibration to reflect the US population <4 y old. This article focuses on food consumption patterns of 0- to 23.9-mo-olds (*n *= 2635). **Supplemental Table 1** shows the unweighted demographic details on the sample compared with the US population.

### Data collection

The full survey instrument comprised a screener questionnaire to identify eligible participants, a recruitment questionnaire consisting of sociodemographic and lifestyle questions (e.g., physical activity, television viewing, and sleep habits), a feeding-practices questionnaire (e.g., breastfeeding practices, introduction of complementary foods), and one 24-h dietary recall (*n *= 3235). A random subsample of 25% (*n *= 799) of the total sampled population provided a second 24-h dietary recall to estimate within-person variance. The instrument was reviewed and approved by the institutional review boards of RTI International, the University of Minnesota Nutrition Coordinating Center, and the Docking Institute of Public Affairs, Fort Hays State University.

The dietary recall interviews were conducted by telephone and administered by certified interviewers using multiple-pass 24-h recall methodology and the Nutrition Data System for Research (NDSR, version 2015: University of Minnesota, Minneapolis, Minnesota). Interviews were conducted with the parent or caregiver primarily responsible for feeding the child, and a form was provided to assist the parent in collecting dietary recall data from daycare centers and other caregivers where the child may have spent part of the day. Only 13% of meals reported were consumed when the child was not with the respondent for part of the 24-h recall period.

### Data analysis

All foods and beverages reported in the 24-h dietary recalls were assigned to food groups developed for the study. These were based on the food-grouping system previously developed for FITS 2008 (35), but updated in 2016 to align with food groups from the USDA's What We Eat in America survey (36) and to account for foods and beverages consumed by infants and young children. Estimation of breast-milk volumes replicated the methods used in previous FITS surveys: for infants <12 mo, these were based on defined amounts by age, adjusted for the total volume of other milks consumed during the recall day; for children aged ≥12 mo, these were based on volume per feeding (35). The estimated percentage of children consuming specific foods or food groups was calculated on the basis of a single 24-h dietary recall, which has been confirmed elsewhere as appropriate for estimation at the population level (37). SAS software (version 9.3; SAS Institute) and SAS-callable SUDAAN software (release 11; RTI International) were used to incorporate sample weights and produce point estimates and SEs that reflect the US population of children aged birth to 47.9 mo.

If there were ≥30 consumers and nonconsumers in each of the 3 largest race/ethnicity groups (Hispanic, NHW, NHB), we performed exploratory analyses (i.e., we did not start with a specific hypothesis) to assess whether consumption patterns differed between race/ethnicity groups. We highlight occurrences where the difference in the percentage consuming a given food between 1 race/ethnicity group (the comparison group) and the other 2 groups is large and the 95% CI for the OR for the comparison group compared with the other 2 groups does not contain the null value of 1.

## Results

In this section, the results are presented first in relation to the general population, followed by selected findings according to racial/ethnic differences. Findings for the percentage of the general population (i.e., regardless of racial/ethnic group) consuming are presented for selected food groups in 3-mo intervals for infants aged <6 mo, and in 6-mo intervals for infants aged ≥6 mo and toddlers. The interested reader may find additional results for the general population in 3-mo intervals for infants >6 mo old and toddlers in **Supplemental Table 2**, and results for more food groups, and by race/ethnicity, in **Supplemental Table 3** for infants (aged 0–11.9 mo) and in **Supplemental Table 4** for toddlers.

### Breastfeeding and breastfeeding alternatives

**FIGURE 1:**
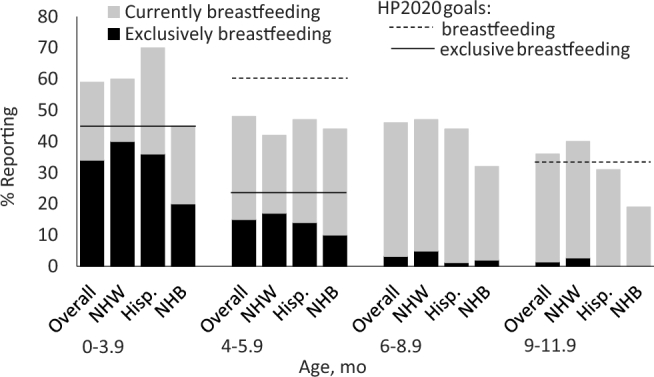
Current and exclusive breastfeeding rates by race and ethnicity, from FITS 2016 feeding practices questionnaire. Lines represent HP 2020 goals (38) for exclusive breastfeeding at ages 3 (0–3.9-mo columns) and 6 (4–5.9-mo columns; solid line) mo and for any breastfeeding at ages 6 (4–5.9-mo columns; dashed line) and 12 (9–11.9-mo columns) mo. No HP 2020 goals are set for breastfeeding at age 9 mo. See Table 1 for sample sizes. FITS, Feeding Infants and Toddlers Study; Hisp., Hispanic; HP, Healthy People; NHB, non-Hispanic black; NHW, non-Hispanic white.

On the basis of responses to the feeding-practices questionnaire, which asked about practices not limited to the day of the recall, ∼85% of 0- to 11.9-mo-olds were ever breastfed (Supplemental Table 1). Nearly 60% of 0- to 3.9-mo-olds were currently breastfeeding at the time of the survey, as well as almost half of 4- to 5.9- and 6- to 8.9-mo-olds and more than one-third of 9- to 11.9-mo-olds (**Figure 1**, **Table 1**). The prevalence of exclusive breastfeeding was much lower than that of current breastfeeding: 34% of 0- to 3.9-mo-olds and 15% of 4- to 5.9-mo-olds and <5% of 6- to 8.9-mo-olds and 9- to 11.9-mo-olds were exclusively breastfed (Figure 1). Findings from the 24-h recall showed that results for the percentage consuming breast milk on the day of the recall were very similar to the percentage currently breastfeeding from the feeding-practices questionnaire: 58% of 0- to 3.9-mo-olds consumed breast milk on the day of the recall, as well as 44% of 4- to 5.9- and 6- to 8.9-mo-olds, 34% of 9- to 11.9-mo-olds, and nearly one-quarter of 12- to 14.9-mo-olds; by 21–23.9 mo, <5% were consuming breast milk (**Table 2**).

**TABLE 1:** Sample sizes by race/ethnicity for infants aged 0–11.9 mo^1^

	Sample size, *n*			
Child's age, mo	Overall	NHW	Hispanic	NHB
0–3.9	305	198	49	38
4–5.9	294	191	47	38
6–8.9	465	318	62	62
9–11.9	430	294	63	51

1 Values are sample sizes corresponding to data presented in Figures 1 (all columns) and 2 (overall column). NHB, non-Hispanic black; NHW, non-Hispanic white.

**TABLE 2:** Consumption of breast milk, infant formula, and cow milk during a single 24-h dietary recall: general population, children aged 0–23.9 mo^1^

	Child's age, mo							
Food group	0–3.9 (*n *= 305)	4–5.9 (*n *= 295)	6–8.9 (*n *= 468)	9–11.9 (*n *= 434)	12–14.9 (*n *= 412)	15–17.9 (*n *= 308)	18–20.9 (*n *= 251)	21–23.9 (*n *= 162)
Breast milk	58	44	44	34	24	12	5.5	4.7
Infant formula	59	69	67	63	12	3.1	1.6	0.5
Any cow milk^2^	1.8	2.0	3.7	17	78	84	83	86
Whole	1.4	1.2	1.5	10	67	71	71	58
Reduced-fat	0	0.7	1.4	5.6	11	10	13	23
Low-fat	0.4	0	0.8	1.5	3.2	3.3	3.5	9.0
Nonfat	0	0.2	0	0.2	1.7	2.4	3.6	2.5

1 Values are mean percentages of children (regardless of race/ethnicity) consuming the food category during a single 24-h recall.

2 Includes all fat levels, as well as flavored, unflavored, or powdered.

Approximately two-thirds of infants (aged <12 mo) consumed infant formula on the day of the survey (Table 2). The percentage consuming was much lower among toddlers, with only 12% of 12- to 14.9-mo-olds and <4% of 15- to 23.9-mo-olds consuming formula. Less than 4% of 0- to 8.9-mo-olds but 17% of 9- to 11.9-mo-olds consumed cow milk (Table 2). Among toddlers, cow milk was consumed by 78–86%, with most consuming whole milk (58–71%), some consuming reduced-fat milk (10–23%), and few consuming low-fat or nonfat milk (<4% except for 12- to 23.9-mo-olds who consumed low-fat milk, 9%) (Table 2).

### Introduction of complementary foods (infants aged 0–5.9 mo)

Seventeen percent of 0- to 3.9-mo-olds and 73% of 4- to 5.9-mo-olds consumed a complementary food on the day of the recall, and those who consumed any formula (regardless of whether they also consumed breast milk) were more likely to consume complementary foods than those who consumed no formula (i.e., exclusively breastfed) (**Table 3**). Few 0- to 3.9-mo-olds who consumed no formula consumed any complementary foods (5%), but 27% of 0- to 3.9-mo-olds receiving formula consumed complementary foods. The same pattern was observed for 4- to 5.9-mo-olds: 85% of infants receiving formula consumed complementary foods compared with 51% of infants not consuming any formula. However, 4- to 5.9-mo-olds receiving formula were less likely to consume potentially non–age-appropriate complementary foods (e.g., bread rolls, pretzels, hot dogs; foods that may be less nutrient dense, contain fewer developmentally important nutrients, or have a texture that is not developmentally appropriate for infants, unlike infant cereal and home-made or commercial puréed foods) compared with those consuming no formula (3.8% compared with 14%, respectively) (Table 3).

**TABLE 3:** Consumption of complementary foods during a single dietary recall by breastfeeding status among children aged 0–5.9 mo^1^

	Children consuming, %					
	Age 0–3.9 mo			Age 4–5.9 mo		
Type of complementary foods	Overall (*n *= 305)	Consumed no formula (*n *= 139)	Consumed any formula^2^ (*n *= 162)	Overall (*n *= 295)	Consumed no formula (*n *= 100)	Consumed any formula^2^ (*n *= 192)
No complementary foods	83	95	73	27	49	15
Any complementary foods	17	5	27	73	51	85
Infant cereal only^3^	5.7	2.3	8.7	13	5.9	17
Puréed baby foods^4^	6.4	0.8	11	53	31	64
Other food or beverage^5^	4.6	1.8	6.9	7.3	14	3.8

1 Values are mean percentages of children consuming the complementary food category during a single 24-h recall.

2 Includes children who consumed both formula and breast milk as well as children who consumed formula and no breast milk.

3 Includes any kind of infant cereal, regardless of grain (i.e., rice, oat, quinoa, wheat, multigrain, or unknown grain).

4 Includes any puréed fruit, vegetable, or meat, whether commercial jarred baby food or homemade puréed fresh foods; child may also consume infant cereal in addition to these but does not consume other (not puréed) foods.

5 Includes any food that is not infant cereal or commercial or homemade puréed baby food.

**FIGURE 2:**
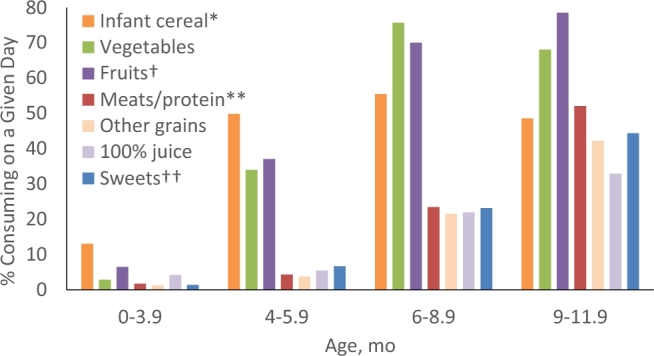
Percentages of infants consuming complementary foods by age, from FITS 2016 24-h dietary recall. *May include other grains; **excludes cheese and yogurt; ^†^excludes fruit juice; ^††^includes sugar-sweetened beverages. See Table 1 for sample sizes. FITS, Feeding Infants and Toddlers Study.

Among 4- to 5.9-mo-olds, regardless of whether they received formula, the most commonly consumed complementary food was iron-fortified infant cereal (50%); few other grains were consumed (**Figure 2**). Baby-food fruits (excluding 100% juice) and baby-food vegetables, respectively, were the second and third most commonly consumed complementary foods in this age group (29% and 27%). Approximately 5% of 4- to 5.9-mo-olds consumed 100% fruit juice. Few 4- to 5.9-mo-olds consumed meats (many of which, such as beef, are iron rich) and other nondairy protein foods (4.3%); the most-commonly consumed sources of protein were other (nonmeat) foods (2.3%), whereas baby-food meats were the least consumed (1.1%) (**Table 4**). However, 6.7% consumed some type of dessert, sweet, or sweetened beverage.

**TABLE 4:** Consumption of complementary foods during a single 24-h dietary recall: general population, children 0–23.9 mo^1^

	Child's age, mo				
Food group	0–3.9 (*n *= 305)	4–5.9 (*n *= 295)	6–11.9 (*n *= 902)	12–17.9 (*n *= 720)	18–23.9 (*n *= 413)
Any grain products	14	54	84	94	96
Infant cereal^2^	13	50	52	16	5.3
Family cereal^3^	0.6	1.1	20	50	58
Baby finger foods^4^	1.1	4.4	33	16	5.7
Any fruit^5^	6.5	37	74	75	79
Any 100% fruit juice^6^	4.2	5.5	27	45	55
Any vegetable^7^	2.9	34	72	73	71
Baby-food vegetables^8^	1.5	27	45	8.7	2.5
Vegetables (not baby food)^9^	0.9	7.6	29	58	60
White potatoes^10^	0.7	2.7	15	33	32
Any meat/other protein food^11^	1.7	4.3	41	88	91
Baby-food meats	0.2	1.1	4.2	0.9	0.6
Meats (not baby food)	1.1	1.3	26	68	72
Other protein sources^12^	0.8	2.3	26	68	75
Any sweets/sugar-sweetened beverage^13^	1.4	6.7	34	73	80
Sugar-sweetened beverages	0.1	2.3	8.5	27	31
Savory snacks^14^	0.4	0.7	77	17	20

1 Values are mean percentages of children (regardless of race/ethnicity) consuming the food category during a single 24-h recall.

2 Includes any kind of baby-food cereal, regardless of grain (i.e., rice, oat, quinoa, wheat, multigrain, or unknown grain).

3 Includes any ready-to-eat or hot cereal that is not infant cereal.

4 Includes pretzels, crackers, rice cakes, and baby-food puffs.

5 Includes commercial and homemade puréed baby-food fruit and non–baby-food fruit; excludes 100% fruit juice.

6 Includes any 100% fruit juice regardless of whether it is specifically labeled for babies or not. Beverages that are <100% fruit juice are included in sugar-sweetened beverages.

7 Includes dark-green, orange, red, starchy, and other vegetables, whether baby food or not, as well as white potatoes.

8 Includes commercial and homemade puréed baby-food vegetables.

9 Includes non–baby-food dark-green, orange, red, starchy, and other vegetables; excludes baby food and white potatoes.

10 Includes fried potatoes, mashed potatoes and mixtures, and baked potatoes.

11 Includes meats and nonmeat sources of protein, including cheese and yogurt.

12 Includes dried beans and legumes; eggs; vegetarian meat substitutes; nuts, nut butters, and seeds; cheese; and yogurt.

13 Includes cakes, pies, chocolate/sweet cookies, bars, brownies, sweet rolls, doughnuts, muffins, and quick breads; frozen desserts, syrups, and sugar; as well as sugar-sweetened beverages.

14 Includes chips, corn chips, popcorn, snack mix, and puffs (non–baby food).

### Consumption patterns of older infants (aged 6–11.9 mo)

Among 6- to 11.9-mo-olds, 84% consumed a grain-based food (Table 4), with iron-fortified infant cereal being the most common (52%), followed by grain-based baby finger foods (33%) and family (i.e., noninfant) cereal (20%). Approximately three-quarters consumed a serving of fruit (74%) or vegetables (72%). The most commonly consumed fruits (excluding 100% fruit juice) before 12 mo were apples, bananas, and pears; and the most commonly consumed vegetables were sweet potatoes, green beans, carrots, squash, and mashed potatoes/other potato mixtures (data not shown). Approximately 27% of 6- to 11.9-mo-olds consumed 100% fruit juice. Among 6- to 11.9-mo-olds, 41% consumed a meat or other protein, but <5% consumed baby-food meats. Nonbaby-food meats and other proteins were about equally likely to be consumed (26%). One-third of 6- to 11.9-mo-olds consumed sweets, including sugar-sweetened beverages (SSBs) (Table 4).

### Consumption patterns of toddlers (aged 12–23.9 mo)

Most toddlers consumed some grain products (>90%), and approximately half consumed family (i.e., not infant) cereal (Table 4). For younger toddlers (aged 12–17.9 mo), more consumed unsweetened cereal than presweetened cereal (30% compared with 23%), but older toddlers (aged 18–23.9 mo) were equally likely to consume presweetened and unsweetened cereal (29% and 27%, respectively; Supplemental Table 4). After cereal, the most-commonly consumed grain-based foods among toddlers were bread, crackers, and rice and pasta (Supplemental Table 4).

Approximately three-quarters of toddlers consumed a fruit on the day of the survey, and a similar percentage consumed a vegetable (Table 4); however, if white potatoes are excluded, only ∼60% consumed a vegetable. Most fruits consumed were fresh fruits (Supplemental Table 4). The most-commonly consumed category of vegetables was white potatoes (∼33%), followed by red and orange vegetables (25%), other starchy vegetables (∼15%), dark-green vegetables (∼12%), and other vegetables (<3%) (Supplemental Table 4). Nearly half of 12- to 17.9-mo-olds and just over half of 18- to 23.9-mo-olds consumed 100% fruit juice (Table 4). The AAP (39) has recently recommended that children aged 1–3 y consume no more than 4 ounces (118 mL) of 100% fruit juice/d, but nearly one-quarter of 12- to 14.9-mo-olds and nearly half of 21- to 23.9-mo-olds exceeded this recommendation on the day of the recall (**Table 5**). At the time of the survey, the AAP recommendation was no more than 6 ounces (177 mL), and 14% of 12- to 14.9-mo-olds and 32% of 21- to 23.9-mo-olds exceeded that amount (Table 5).

**TABLE 5:** Consumption of 100% fruit juice in excess of 2017 AAP-recommended amount of 4 ounces (118 mL) or 2001 AAP-recommended amount of 4–6 ounces (118–177 mL)/d in children aged 12–23.9 mo^1^

	Child's age, mo			
Amount consumed	12–14.9 (*n *= 412)	15–17.9 (*n *= 308)	18–20.9 (*n *= 251)	21–23.9 (*n *= 162)
>4 ounces (>118 mL)	23 ± 2.9	31 ± 3.6	40 ± 4.0	46 ± 4.7
>6 ounces (>177 mL)	14 ± 2.2	23 ± 3.3	29 ± 3.8	32 ± 4.6

1 Values are mean ± SE percentages of children consuming more than the 2017 AAP-recommended 4 ounces (118 mL) of juice/d (39) or the 2001 AAP recommendation (40) [reaffirmed in 2006 (41) and in place at the time of the survey] of 4–6 ounces (118–177 mL)/d, during a single 24-h recall. AAP, American Academy of Pediatrics.

Approximately two-thirds of toddlers consumed meats, and about the same percentage consumed nonmeat protein foods (Table 4). The most-commonly consumed meat among both younger and older toddlers was chicken or turkey (39% of 12- to 17.9-mo-olds and 42% of 18- to 23.9-mo-olds), but cured meats were also consumed by more than one-quarter of toddlers (Supplemental Table 4). Beef lagged farther behind at just over 10%. The most commonly consumed nonmeat protein foods consumed by toddlers were cheese, eggs, yogurt, and nuts and nut butters (Supplemental Table 4).

Approximately three-quarters of toddlers consumed sweets and SSBs (Table 4). The most-commonly consumed solid sweets (i.e., not an SSB) were sweet bakery items (27% of 12- to 17.9-mo-olds and 36% of 18- to 23.9-mo-olds) and sugars, syrups, preserves, and jellies (22% of 12- to 17.9-mo-olds and 30% of 18- to 23.9-mo-olds; Supplemental Table 4). One-quarter to one-third of toddlers consumed an SSB on the day of the survey (27% of 12- to 17.9-mo-olds and 31% of 18- to 23.9-mo-olds; Table 4).

### Notable differences by race/ethnicity

#### Infants (aged 0–11.9 mo)

Throughout the first year, NHB children were less likely than NHW and Hispanic infants to consume breast milk on the day of the recall (43% compared with 53–59%—OR: 0.63; 95% CI: 0.36, 1.12 for ages 0–5.9 mo; 22% compared with 36–42%—OR: 0.43; 95% CI: 0.26, 0.71 for ages 6–11.9 mo; **Table 6**). NHB 0- to 3.9-mo-olds and 4- to 5.9-mo-olds also had the lowest rates of exclusive breastfeeding (20% and 10%, respectively; Figure 1). Several notable differences by race and ethnicity were observed for specific grain products: NHB 6- to 11.9-mo-olds were much more likely than their NHW and Hispanic counterparts to consume baby-food puffs (14% compared with 27%; OR: 0.43; 95% CI: 0.24, 0.77) and ready-to-eat cereal (5% compared with 13–16%; OR: 0.33; 95% CI: 0.14, 0.77) (Table 6). Few other notable differences by race/ethnicity were observed for infants.

**TABLE 6:** Consumption of selected foods during a single 24-h dietary recall, by race/ethnicity, in children aged 0–23.9 mo^1^

	Consumers,^2^ %				
Food group	Hispanic	NHW	NHB	Comparison group^3^	OR (95% CI)^4^
Age 0–5.9 mo					
* n*	96	389	76	—	—
Breast milk	59	53	43	NHB	0.63 (0.36, 1.12)
Age 6–11.9 mo					
* n*	125	612	113	—	—
Breast milk	36	42	22	NHB	0.43 (0.26, 0.71)
RTE cereal^5^	16	13	5.3	NHB	0.33 (0.14, 0.77)
Baby-food puffs	27	27	14	NHB	0.43 (0.24, 0.77)
Age 12–23.9 mo					
* n*	161	770	150	—	—
Breast milk	11	13	5	NHB	0.36 (0.18, 0.72)
Sugar-sweetened beverages	28	25	45	NHB	2.31 (1.50, 3.57)
White potatoes^6^	28	30	46	NHB	2.03 (1.25, 3.30)
Cheese	34	43	27	NHB	0.55 (0.35, 0.89)
Rice and pasta	35	20	25	Hispanic	1.88 (1.06, 3.32)
Dried beans, peas, legumes	14	9	8	Hispanic	1.84 (1.06, 3.20)
Eggs and egg dishes	30	21	24	Hispanic	1.74 (1.14, 2.64)
Sweet bakery^7^	22	36	37	Hispanic	0.51 (0.31, 0.82)
Any fruit^8^	70	78	63	NHW	1.72 (1.20, 2.48)
Orange and red vegetables	20	26	15	NHW	1.59 (1.08, 2.32)
Any 100% juice^9^	55	37	56	NHW	0.48 (0.35, 0.67)

1 NHB, non-Hispanic black; NHW, non-Hispanic white; RTE, ready-to-eat.

2 Values are mean percentages of children consuming the food category during a single 24-h recall unless otherwise indicated.

3 The comparison group was the group with the highest or lowest percentage consuming among the 3 groups that was also the most different from the value of the middle group. For example, for dried beans, peas, and legumes, the group with the highest percentage consuming is Hispanic and the lowest is NHB; we chose Hispanic for the comparison group because the middle value (9%, NHW) is much closer to the value for NHB (8%) than the value for Hispanic (14%).

4 Values are ORs for the percentage consuming in the comparison group compared with the percentage consuming for the other 2 groups, and the 95% CI around the OR.

5 Includes any RTE cereal that is not infant cereal.

6 Includes fried potatoes, mashed potatoes and mixtures, and baked potatoes.

7 Includes cakes, pies, chocolate/sweet cookies, bars, brownies, sweet rolls, doughnuts, muffins, and quick breads.

8 Includes any fruit whether baby food or not; excludes 100% fruit juice.

9 Includes any 100% fruit juice regardless of whether it is specifically labeled for babies or not. Beverages that are <100% fruit juice are included in sugar-sweetened beverages.

#### Toddlers (aged 12–23.9 mo)

Beyond the first year, NHB toddlers continue to be less likely than NHW and Hispanic toddlers to consume breast milk (5% compared with 11–13%; OR: 0.36; 95% CI: 0.18, 0.72). In addition, NHB toddlers were more likely to consume SSBs (45% compared with 25–28%; OR: 2.31; 95% CI: 1.50, 3.57) and white potatoes (46% compared with 28–30%: OR: 2.03; 95% CI: 1.25, 3.30) than NHW and Hispanic toddlers and less likely to consume cheese (27% compared with 34–43%: OR: 0.55; 95% CI: 0.35, 0.89). Hispanic toddlers were almost twice as likely to consume rice and pasta (35% compared with 20–25%; OR: 1.88; 95% CI: 1.06, 3.32), eggs (30% compared with 21–24%; OR: 1.74; 95% CI: 1.14, 2.64), and dried beans and legumes (14% compared with 8–9%; OR: 1.84; 95% CI: 1.06, 3.20) than non-Hispanic toddlers (Table 6) and less likely to consume sweet bakery items (22% compared with 36–37%; OR: 0.51; 95% CI: 0.31, 0.82). NHW toddlers were more likely to consume fruit than NHB and Hispanic toddlers (78% compared with 63–70%; OR: 1.72; 95% CI: 1.20, 2.48) and red and orange vegetables (26% compared with 15–20%; OR: 1.59; 95% CI: 1.08, 2.32) and less likely to consume 100% fruit juice (37% compared with 55–56%; OR: 0.48; 95% CI: 0.35, 0.67). No notable differences were seen in the percentage of toddlers consuming any meats by race and ethnicity.

## Discussion

The large sample of children <24 mo old studied in FITS 2016 provides the best current estimates of infant and toddler food and nutrient intake and allows us to make some comparisons by race and ethnicity. Several positive findings suggest that policy and public health initiatives may be changing infant and toddler feeding practices to better align with early-feeding recommendations. However, there is some evidence that these improvements have not been consistent. To put the results of FITS 2016 in context, we have compared our results with previously published data from past FITS surveys and other relevant studies in the following discussion. The comparisons to previous FITS surveys are not a statistically rigorous trend analysis in that they do not account for changes in food-group organization (which has evolved with each study iteration) or control for changes in the demographic characteristics of the US population over time.

### Breastfeeding

There appears to have been a trend for both the initiation and duration of breastfeeding to have increased in the total population over the 3 FITS surveys. In 2016, 83% of 4- to 23.9-mo-olds were ever breastfed, compared with 80% in FITS 2008 and 76% in 2002 (32). The percentage of infants currently breastfed was also modestly higher than in 2008 for 4- to 5.9-mo-olds (42% in 2008, 48% in 2016) and 6- to 8.9-mo-olds (37% in 2008, 46% in 2016), and remained similar for 9- to 11.9-mo-olds (37% in 2008, 36% in 2016) (32). In addition, in FITS 2016, 2 of the Healthy People 2020 goals for breastfeeding (38) were met in the total population: the percentage of mothers initiating breastfeeding (Healthy People goal is 81.9%; result from FITS 2016 is 83%) and continuing to breastfeed at 12 mo (Healthy People goal is 34.1%; result from FITS 2016 is 36%). This success may be attributable to changing norms and cultural shifts in preferences to prolong nursing, as well as interventions intended to promote increased breastfeeding, such as the Baby Friendly Hospital Initiative (42). For example, 21% of US births in 2016 occurred in Baby Friendly facilities, compared with 3% in 2007.

Despite these gains, the prevalence and duration of exclusive breastfeeding still fall short of Healthy People 2020 goals (38) and AAP recommendations for exclusive breastfeeding until ∼6 mo and continued breastfeeding until ∼1 y (9). In addition, notable differences in breastfeeding behavior still exist by race and ethnicity. Although more infants overall continued to breastfeed in later infancy than in 2008, the prevalence was lower among minorities. Across all age categories, the prevalence of breastfeeding among NHB mothers was lower than the prevalence of breastfeeding among the total population. The prevalence of exclusive breastfeeding up to 6 mo was appreciably lower for NHB infants than for NHW and Hispanic infants. As such, the progress in overall breastfeeding initiation and the moderate success in extending breastfeeding duration mask important racial/ethnic differences and emphasizes the need to address these disparities. Previous research has shown that ∼60% of women stop breastfeeding earlier than they would like (43). Known barriers to breastfeeding include lack of family, peer, and health provider support; lack of social/cultural acceptance; inadequate knowledge; preference to bottle feed; difficulties around initiation (e.g., latching problems, breast discomfort); and the need to return to work (24, 44). Mothers who returned to work within 6 wk postpartum were 3 times as likely to stop breastfeeding before the recommended 6 mo, after adjusting for race, income, and education (45). Inequities in paid parental leave may further contribute to the breastfeeding disparity seen among low-income and racial minority groups (46).

### Complementary foods

FITS 2016 also provides evidence of success in complementary feeding practices, such as timing of introduction of complementary foods, while signaling the need for improvement in others, such as low consumption of iron-rich foods, fruits, and vegetables and excess consumption of 100% fruit juice and SSBs.

#### Age of introduction of complementary foods

Similar to findings from NHANES 2009–2012 (47), the prevalence of the early introduction of complementary foods continues to decline, with only 17% infants <4 mo old being introduced to foods other than breast milk or formula in 2016. However, the introduction of complementary foods before 4 mo of age was considerably higher among infants who consumed any formula than among those who consumed no formula. The age of introduction of complementary foods and its association with overweight and obesity in later childhood remains equivocal, with only a few studies showing that early introduction before 4 mo of age may be associated with a higher BMI, whereas the majority of studies failed to show a relation between the age of introduction of complementary foods and later adiposity in childhood (5, 48, 49).

#### Consumption of iron-rich foods

Sixteen percent of 1- to 2-y-olds were reported in 2010 to be iron deficient (50), and evidence from longitudinal studies highlights some of the long-term cognitive impairments associated with iron deficiency in early childhood (51). Iron deficiency is caused by multiple factors, including insufficient intake, genetic factors affecting iron metabolism, and excessive losses. Both globally and in the United States, iron deficiency is common in young children (52). The AAP recommends that full-term infants who are partially or exclusively breastfed receive ∼1 mg Fe ⋅ kg^−1^ ⋅ d^−1^ starting at 4 to 6 mo, in the form of a liquid iron supplement, until iron-rich foods like iron-fortified infant cereal and iron-rich meats are introduced (50). In FITS 2008, 12% of 6- to 11.9-mo-olds consumed less than the Estimated Average Requirement for iron, an increase from 7% in FITS 2002 (53). Elsewhere in this supplement issue, FITS 2016 data show that figure has increased to 18% (52). In addition, among 6- to 11.9-mo-olds, only 15% consumed any type of supplement and <5% consumed iron supplements specifically (52). Iron-fortified infant cereal is often thought of as the first solid food introduced to infants. However, the consumption of iron-fortified infant cereal declined since 2008: 55% of 6- to 8.9-mo-olds in 2016 (Supplemental Table 2) compared with 82% and 79% in 2002 and 2008, respectively (32). This decline was not compensated for by the consumption of iron-rich puréed baby-food meats, which was also low and peaked at 5% among 6- to 8.9-mo-olds (Supplemental Table 2).

Another compounding contributor to iron deficiency in this age group is the early introduction and consumption of cow milk, which has been associated with gastrointestinal blood loss in infants from conditions such as food protein–induced allergic proctocolitis and cow-milk protein allergy (54, 55). The AAP recommends that parents avoid the introduction of cow milk before 12 mo (56). The prevalence of cow-milk consumption earlier than 12 mo remained at ∼17% of 9- to 11.9-mo-olds compared with FITS 2008 (32); however, those infants consuming cow milk tended to consume large quantities. These observations highlight the need for the continued education of caregivers about the avoidance of cow milk in the first year of life and the need for efforts to educate parents and caregivers of the sources and importance of iron in their child's diet.

#### Fruits, 100% fruit juice, and vegetables

Fruit and vegetable consumption for infants <9 mo old continued to increase or remain level. In FITS 2016, 70% of 6- to 8.9-mo-olds and 79% of 9- to 11.9-mo-olds consumed any fruit (Supplemental Table 2) compared with 65% and 81% in FITS 2008 (32), and 76% of 6- to 8.9-mo-olds and 78% of 9- to 11.9-mo-olds consumed any vegetable (Supplemental Table 2) compared with 63% and 72% in FITS 2008 (32). For toddlers, ∼80% consumed a fruit [a decrease somewhat from 85–90% in 2008 (32)] and ∼70% consumed a vegetable [about the same as in 2008 (32)]. Nonetheless, as with findings in NHANES (47) and FITS 2008 (32), the amount and variety of fruit and vegetable consumption remained far below recommendations in many older infants, and fell even shorter in toddlers. The AAP recommends offering a fruit or vegetable at every meal and snack after 6 mo (57). However, >20% of 12- to 23.9-mo-olds did not consume a single serving of fruit on the day of recall, and 30% did not consume any vegetable servings.

Several areas for improvement were noted with regard to types of fruits and vegetables consumed among 12- to 23.9-mo-olds. Although toddlers were about as likely to consume a serving of vegetable (71–74%; Supplemental Table 2) as a serving of fruit (71–81%), only ∼60% consumed a vegetable other than white potatoes, and approximately one-third consumed white potatoes.

Current studies have linked the consumption of fruits and vegetables to a decreased risk of several chronic diseases and obesity (15, 58–69), and early feeding preferences may persist throughout the life span (70). Innovative approaches to introduce and sustain the consumption of a variety of fruits and vegetables should be a high priority, not just for infant feeding but also for families.

In 2017, the AAP updated its position on fruit juice and recommended delaying the introduction of juice until 12 mo and limiting the maximum amount of juice for young toddlers to no more than 4 ounces (118 mL) of 100% fruit juice/d (58, 71, 72). However, at the time of the survey, the previous recommendation from the AAP allowed for no more than 4–6 ounces (118–177 mL) daily starting in late infancy. A recent meta-analysis concluded that the consumption of 100% fruit juice was associated with a small amount of excess weight gain in childhood (58). We found that infants and toddlers were somewhat less likely to consume 100% fruit juice in 2016 than in FITS 2008 (32), with the greatest decrease between 6 and 12 mo [e.g., for 6- to 11.9-mo-olds, the percentage consuming 100% juice fell from 31–41% in 2008 (32) to 22–33% in 2016; Supplemental Table 2]. However, one-third of 9- to 11.9-mo-olds still drank 100% fruit juice (Supplemental Table 2), which is not consistent with the most recent AAP recommendations to delay the introduction of fruit juice until 12 mo (39). Furthermore, nearly one-quarter of the youngest toddlers (12–14.9 mo) were consuming more than the new recommended maximum amount of 4 ounces (118 mL) of 100% fruit juice/d, and 14% were consuming more than the previously recommended maximum amount of 6 ounces (177 mL) (40). Nearly half of toddlers aged 21–23.9 mo were consuming >4 ounces (118 mL) and nearly one-third were consuming >6 ounces (177 mL)/d.

#### SSBs

The consumption of SSBs has increased for some age groups and decreased for others since 2008. In 2016, few younger infants (<6 mo) consumed SSBs, but among 9- to 11.9-mo-olds, the percentage consuming increased from 11% in 2008 (32) to 14% in 2016 (Supplemental Table 2) and doubled—from 14% in 2008 (32) to 28% in 2016 (Supplemental Table 2)—for 12- to 14.9-mo-olds. Peak consumption in 2016 was in 18- to 20.9-mo-olds (33% consuming an average of 106 kcal/consumer; Supplemental Table 2). Clearly, there is an opportunity to further delay the introduction of SSBs and to reduce consumption in late infancy and toddlerhood. There were racial/ethnic disparities in the consumption of SSBs: NHB toddlers were more likely to consume SSBs than NHW and Hispanic toddlers. Sugary drinks should be avoided because they contribute calories but few nutrients and may decrease the child's appetite for more nutritious foods. Moreover, the consumption of high-caloric-density foods and beverages in early childhood significantly increases the likelihood of consumption of these products later in childhood, increases the risk of dental caries, and may contribute to the prevalence of early childhood obesity (15, 60, 62, 63).

### Study strengths and limitations

Data from the FITS 2016 can make an important contribution to early-childhood nutrition. The study provides population-level data and builds on previous FITS studies, which, in turn, can help us to monitor the diets of very young children in the United States. It can allow us to compare feeding practices to national recommendations and also contribute to new dietary guidelines for the population aged from birth to 2 y.

A potential limitation of the FITS 2016 study applies to all cross-sectional studies that use self-reported 24-h recall data, because they are subject to reporting bias and errors in remembering what and how much was consumed. Some of the race/ethnicity differences in food-consumption patterns that we found may be due to household economic and education status, access to food (related to geographic location; i.e., food deserts), and other social determinants rather than, or in addition to, race/ethnicity. Previous research has yielded conflicting conclusions about the role of race/ethnicity on breastfeeding. One study found that maternal education had a greater effect on breastfeeding incidence than race/ethnicity (73), whereas another reported that, even after accounting for socioeconomic status, NHBs were consistently less likely to breastfeed than were NHWs (74, 75). The analysis of differences between racial/ethnic groups was exploratory, because previous FITS studies did not present results by race/ethnicity, and we did not have any specific hypotheses we were testing. However, the race/ethnicity differences we report are largely consistent with the limited data available in the literature (47) and suggest that although national data are useful for documenting trends, it is critical that they take into account race/ethnicity during study design, data collection, and analysis. Future analyses are planned and include examining racial/ethnic differences in the FITS 2002 and 2008 surveys (informed by the results reported here) and conducting in-depth trend analyses.

### Opportunities for future research

The most recent data from the FITS appear to indicate that we have found some improvements in breastfeeding practices and early introduction of complementary foods; however, several areas of concern remain. These include exclusive breastfeeding; timing of introduction of complementary foods, particularly in formula-fed infants; low consumption of iron-rich foods; early introduction of cow milk; inadequate quantity and variety of fruits and vegetables consumed; and early introduction and consumption of SSBs and 100% fruit juice in the first year of life. In addition, the racial/ethnic disparities in feeding practices highlighted herein may herald disparities in obesity and metabolic disorders later in life (22, 24, 76–78).

Factors that account for the departure from the feeding practices recommended by the AAP remain uncertain. The design of FITS 2016 and the substantial data collection necessary to describe patterns of food consumption limit somewhat our ability to measure and understand the underlying attitudes, beliefs, socioeconomic, and potential environmental factors that led to these patterns. Associated lifestyle factors that may influence consumption patterns will be explored in subsequent publications. Although dietary counseling by primary care providers is a part of routine care, the quality and effectiveness of such counseling may need to be strengthened to be inclusive of cultural preferences, practices, and access to nutritious and affordable foods. To our knowledge, the frequency, quality, and efficacy with which counseling routinely addresses practices such as avoiding the early introduction of solids or cow milk have not been carefully examined (79). Furthermore, we are not aware of any studies that have effectively addressed the substantial intake of high-caloric-density foods that may lead to the development of early obesity. FITS 2016 offers many opportunities to fill further data gaps in relation to the feeding of infants and toddlers. Future exploration of the meal, snack, and beverage patterns in this age group is planned, and it is hoped will shed light on potential obesogenic dietary patterns. In addition, examining food sources of nutrients could provide more insights into how foods selected by caregivers contribute to nutrient intakes, and allow for exploration of differences by race and ethnicity, WIC status, or socioeconomic status. Ongoing surveillance of early-childhood food and beverage consumption that accounts for race and ethnicity is needed to identify feeding patterns that may lead to future health problems early enough to intervene and change behaviors.

Across race and ethnicity, our results are largely consistent with the limited data available in the literature (47) and underline the need for focused education and policy changes to decrease some of the disparities observed in feeding practices and dietary intakes. Whether the differences in food-consumption patterns reflect cultural preferences, choices directed by access to food or food insecurity, or other social justice issues remains uncertain. These differences could also be due to household economic and education status rather than race and ethnicity. Our observations point to the need to tailor education and feeding guidance to specific racial and ethnic groups.

### Conclusions

FITS 2016 provides the most recent cross-sectional estimates of food and beverage consumption for young children in the United States. The sample size of FITS permits exploration of racial/ethnic comparisons that have not previously been described, and although initial findings with regard to differences by race/ethnicity herein are exploratory, our data may set the stage for further research and provide important insights into guidance and interventions that are still needed in order to improve diets.

## Supplementary Material

Supplement Files

## Notes

Published in a supplement to *The Journal of Nutrition*. Select contents of this supplement were presented at the Experimental Biology 2017 conference in a session titled, “Informing B-24 Dietary Guidelines: Findings from the New Feeding Infants and Toddlers Study 2016,” held in Chicago, IL, April 22, 2017. Supplement Coordinators were William Dietz, Milken School of Public Health at the George Washington University and Andrea S Anater, RTI International. The FITS research was funded by Nestlé Research (Lausanne, Switzerland) through a contract with RTI International and its subcontractor, the University of Minnesota. Dr. Dietz is a compensated consultant to RTI on this project. The article contents are the responsibility of the authors and do not necessarily represent the opinions or recommendations of Nestlé. Publication costs for this supplement were defrayed in part by the payment of page charges. This publication must therefore be hereby marked “advertisement” in accordance with 18 USC section 1734 solely to indicate this fact. The opinions expressed in this publication are those of the authors and are not attributable to the sponsors or the publisher, Editor, or Editorial Board of *The Journal of Nutrition*.

Supported by Nestlé Research, Lausanne, Switzerland.

Supplemental Tables 1–4 are available from the “Supplementary data” link in the online posting of the article and from the same link in the online table of contents at https://academic.oup.com/jn/.

Author disclosures: AAR, DJC, ACL, ASA, no conflicts of interest. EFJ is employed by Nestlé Research (funding source); RC is employed by Nestlé Nutrition. WHD has served on the Weight Watchers Scientific Advisory Committee and has received grant support from Bridgespan for analyses of NHANES.

Abbreviations used:

AAP: American Academy of Pediatrics
B-24: birth to 24 mo
FITS: Feeding Infants and Toddlers Study
NHB: non-Hispanic black
NHW: non-Hispanic white
SSB: sugar-sweetened beverage
