# Supplementary material for: Food Consumption Patterns of Infants and Toddlers: Findings from the Feeding Infants and Toddlers Study (FITS) 2016
Source: J Nutr. 2018 Aug 31;148(Suppl 3):1525S–1535S. doi: 10.1093/jn/nxy171 (PMC6126630; doi:10.1093/jn/nxy171)
Supplement: Supplement Files [file nxy171_supplement_files.zip › nut264945-file001.docx]

# Food consumption patterns of Infants and Toddlers: Findings from The Feeding Infants and Toddlers study (FITS) 2016^[[1]](#footnote-2),^^[[2]](#footnote-3),^^[[3]](#footnote-4),^^[[4]](#footnote-5)^

Amira A. Roess,^a^ Emma F. Jacquier,^b^ Diane J. Catellier,^c^ Ryan Carvalho,^d^ Anne C. Lutes,^c^ Andrea S. Anater,^c^ William H. Dietz^a^

^a^ The George Washington University, Milken Institute School of Public Health, 950 New Hampshire Ave, NW, Washington, DC 20052, USA

^b^ Nestlé Research Center, Route du Jorat 57, Vers-chez-les-Blanc, 1000 Lausanne, Switzerland

^c^ RTI International, P.O. Box 12194, Research Triangle Park, NC 27709, USA

^d^ Nestlé Nutrition, 12 Vreeland Rd, Florham Park, NJ 07962, USA

**Corresponding Author:** Amira Roess, The George Washington University, Milken Institute School of Public Health, 950 New Hampshire Ave, 4th Floor, NW, Washington, DC 20052, USA. Phone: 202/994-3799; fax: 202/994-0082; email: [aroess@gwu.edu](mailto:aroess@gwu.edu)

**Author Last Names for PubMed Indexing:** Roess, Jacquier, Catellier, Carvalho, Lutes, Anater, Dietz

**Word Count:** 5948

**Number of Figures:** 2

**Number of Tables:** 6

**Online Supporting Material:** 4 tables

**Running Title:** **Running Title:** FITS 2016 Consumption Patterns of Infants and Toddlers

# Abstract

**Background.** Prevalence of obesity and type 2 diabetes continues to rise. These conditions disproportionately affect minorities and are associated with poor nutrition early in life. Current food consumption patterns can inform pending dietary guidelines for infants and toddlers.

**Objective.** The aim of this study was to describe infant feeding, complementary feeding, and food and beverage consumption patterns of 0- to 23.9-mo-olds in the general population.

**Methods.** The Feeding Infants and Toddlers Study 2016 is a cross-sectional survey of care-givers of children <4y. Dietary data were collected from a national random sample using a 24-h dietary recall (*n*=3,235). Percent consuming was calculated for >400 food groups. Differences in percent consuming between Hispanic, non-Hispanic white (NHW), and non-Hispanic black (NHB) children 0-23.9 mo-old were evaluated using odds ratios and 95 percent confidence intervals.

**Results.** 83% of 0-23.9-mo-olds (*n*=2,635) were ever breastfed; 34% of 0- to 3.9-mo-olds (*n*=305) and 15% of 4- to 5.9-mo-olds (*n*=295) were exclusively breastfed; and 24% of 12- to 14.9-mo-olds (*n*=412) consumed breast milk on the day of the recall. Complementary foods were more likely to be introduced before 4 mo in formula-fed infants (27%) than infants consuming no formula (5%). Half of 4- to 5.9-mo-olds consumed iron-fortified infant cereal, but few consumed iron-rich meats. Among toddlers (12-23.9 mo; n=1,133), over 20% consumed no serving of fruit or vegetable on the day of the recall; about half consumed 100% fruit juice; and a quarter to a third consumed a sugar sweetened beverage.

**Conclusions.** Breastfeeding initiation and duration have improved, but exclusivity remains low. Low consumption of iron-rich foods, fruits, and vegetables and lack of variety in vegetable consumption are problems. Efforts to reduce consumption of SSBs and 100% fruit juice are warranted in early childhood.

**Keywords:** Feeding Infants and Toddlers Study, FITS 2016, food intakes, breastfeeding, eating habits, young children, disparities

# Introduction

Approximately 8% of US infants under 2 years old have high weight relative to recumbent length ([1](#_ENREF_1)), and 14% of 2- to 5-year-olds have obesity ([2](#_ENREF_2)). Obesity at age 5 is a strong predictor of obesity in early adolescence ([3](#_ENREF_3)). Breastfeeding is associated with lower risk for later development of overweight, obesity, and Type 2 diabetes ([4](#_ENREF_4), [5](#_ENREF_5)); however, some studies have questioned the association between breastfeeding and lower risk of other health outcomes, such as cholesterol and blood pressure ([4](#_ENREF_4), [6](#_ENREF_6)). The relationship between age of introduction of complementary foods, however, and later development of overweight and obesity is not consistent ([5](#_ENREF_5)). Identification of the foods and dietary behaviors that may contribute to excess caloric intake can offer insights into dietary strategies to prevent obesity and other poor health outcomes. In addition, previous studies have shown that low-income and some minority populations are disproportionately affected by these poor health outcomes ([7-12](#_ENREF_7)). Consequently, an exploratory consideration of potential racial/ethnic differences in dietary intake may help to guide further research and policy discussions to support more vulnerable populations.

The American Academy of Pediatrics (AAP) has provided comprehensive recommendations for early feeding practices for children under 2 years old. Optimal breastfeeding behaviors recommended by the AAP include exclusive breastfeeding for the first 6 mo of life and continued breastfeeding until at least 12 mo ([9](#_ENREF_9)). Breastfeeding reduces the risks of sudden infant death syndrome, infectious diseases, asthma, obesity, and Type 2 diabetes ([13-18](#_ENREF_13)), all of which disproportionately affect minority children ([12](#_ENREF_12), [19-21](#_ENREF_19)). Model estimates of the excess disease cases, deaths, and costs attributable to suboptimal breastfeeding are substantial for both mothers and infants in the US. For example, estimates are that optimal breastfeeding could prevent over 700 child deaths in the US annually ([22](#_ENREF_22)). National breastfeeding rates remain low, particularly the rate of exclusive breastfeeding in the first 6 mo of life ([23](#_ENREF_23)). Furthermore, low-income minority women, especially non-Hispanic black (NHB) mothers, breastfeed at lower rates than their non-Hispanic white (NHW) counterparts ([24](#_ENREF_24), [25](#_ENREF_25)).

The AAP Committee on Nutrition recommends the introduction of complementary foods between 4 and 6 mo ([26](#_ENREF_26), [27](#_ENREF_27)). At this age, breastmilk alone is typically no longer sufficient to meet an infant’s nutritional requirements without the introduction of other foods and liquids (complementary foods). The introduction of complementary foods represents a critical transition from the largely milk-based infant diet to a diet primarily based on the foods eaten by the rest of the family by the time the child is 18–24 mo old ([28](#_ENREF_28)). The choice of complementary foods may affect intake of essential nutrients that older infants and toddlers may under-consume, such as iron and Vitamin D, as well as nutrients that are often consumed to excess, such as sodium. Small studies have reported that complementary feeding practices are more likely to depart from the AAP recommendations for minority children ([29](#_ENREF_29)), but only limited nationally representative data are available to adequately quantify these differences ([30](#_ENREF_30)).

Despite the links between early nutrition and health outcomes, national data on early feeding practices are largely absent from the literature. Furthermore, the *Dietary Guidelines for Americans*, first issued in 1980, have not yet covered children aged 0–24 mo (the so-called “Birth to 24” or “B-24” age group). However, the first B-24 recommendations are expected to be released in the 2020–2025 *Dietary Guidelines for Americans* ([31](#_ENREF_31)).

The Feeding Infants and Toddlers Study (FITS) is a unique survey that provides national estimates of early feeding behaviors, including breastfeeding and introduction of complementary foods, which can inform the development of dietary guidelines for the B-24 age group. Previous FITS studies reported that the prevalence of children “ever breastfed” was 76+1.1% in 2002 and modestly higher in 2008 (80+1.5%). Those studies also report that infant cereal was introduced between 4 and 6 mo for only 65% of infants in 2002 and 50% in 2008 ([32](#_ENREF_32)). Consumption of fruits and vegetables was substantially lower than recommendations in previous studies as well, with 15–30% of infants and toddlers over 6 mo old consuming no fruit and 25–30% consuming no vegetables on the day of the survey in both FITS 2002 and 2008 ([32](#_ENREF_32)).

The aim herein is to provide a cross-sectional description of the breastfeeding practices, use of human milk substitutes, and complementary feeding behaviors among 0- to 23.9-mo-olds from the FITS 2016. In addition, exploratory analyses were performed to identify potentially important racial/ethnic differences. These results can inform the development of US dietary guidelines for children under 2y and lead to recommendations to optimize both breastfeeding and complementary feeding practices among the general population and, potentially, minorities. In addition, these findings can be used to indicate avenues for future research, such as the development of hypotheses to explore in the analysis of FITS 2002, 2008 and 2016 to compare trends over time.

# Methods

## FITS Survey Methods

A detailed report of the FITS 2016 design and methodology is reported elsewhere in this supplement ([33](#_ENREF_33)). Briefly, the FITS 2016 is a national cross-sectional survey designed to collect data on the food and beverage intake, using the 24-h recall method among children aged under 4 years old living in the 50 states and Washington DC. This method enables subsequent estimation of food and nutrient intakes on the day of the survey. Details of the questionnaire development and testing, sampling, and data collection methodology largely replicate the methods used in previous FITS surveys conducted in 2008 and 2002 ([34](#_ENREF_34), [35](#_ENREF_35)), with some updates to the food classification scheme. The questionnaires from 2008 were used in 2016 with minor modifications and the addition of some new data items (none of which are reflected in this paper). The sample for FITS 2016 was identified through stratified random sampling from four sampling frames to obtain target sample sizes in cells defined by 12 age groups. In addition, a secondary objective of the target sample frames was to obtain adequate cell sizes within each of the 12 age groups of children participating in the Special Supplemental Nutrition Program for Women, Infants and Children (WIC). Sampling weights were calculated to account for the probability of household selection and then adjusted for nonresponse and incomplete coverage by calibration to reflect the US population under 4 years old. This paper focuses on food consumption patterns of 0- to 23.9-mo-olds (*n*=2,635); Supplemental Table 1 shows the unweighted demographic details about the sample compared to the US population.

## Data Collection

The full survey instrument comprised a screener questionnaire to identify eligible participants, a recruitment questionnaire consisting of sociodemographic and lifestyle questions (e.g., physical activity, TV viewing, and sleep habits), a feeding practices questionnaire (e.g., breastfeeding practices, introduction of complementary foods), and one 24-h dietary recall (*n*=3,235). A random subsample of 25% (*n*=799) of the total sampled population provided a second 24-h dietary recall to estimate within-person variance. The instrument was reviewed and approved by the institutional review boards of RTI International; the University of Minnesota Nutrition Coordinating Center; and the Docking Institute of Public Affairs, Fort Hays State University.

The dietary recall interviews were conducted by telephone and administered by certified interviewers using multiple-pass 24-h recall methodology and the Nutrition Data System for Research (NDSR, version 2015: University of Minnesota, Minneapolis, MN). Interviews were conducted with the parent or caregiver primarily responsible for feeding the child, and a form was provided to assist the parent in collecting dietary recall data from daycares and other caregivers where the child may have spent part of the day. Only 13% of meals reported were consumed when the child was not with the respondent for part of the 24-h recall period.

## Data Analysis

All foods and beverages reported in the 24-h dietary recalls were assigned to food groups developed for the study. These were based on the food grouping system previously developed for FITS 2008 ([35](#_ENREF_35)), but updated in 2016 to align with food groups from the USDA’s What We Eat in America survey ([36](#_ENREF_36)) and to account for foods and beverages consumed by infants and young children. Estimation of breastmilk volumes replicated the methods used in previous FITS surveys: for infants under 12 months, these were based on defined amounts by age, adjusted for the total volume of other milks consumed during the recall day; for children 12 mo and older, these were based on volume per feeding ([35](#_ENREF_35)). The estimated percentage of children consuming specific foods or food groups was calculated based on a single 24-h dietary recall, which has been confirmed elsewhere as appropriate for estimation at the population level ([37](#_ENREF_37)). SAS software (version 9.3, SAS Institute, Cary, NC) and SAS-callable SUDAAN^®^ software (Release 11, RTI International: Research Triangle Park, NC) were used to incorporate sample weights and produce point estimates and standard errors that reflect the US population of children aged birth to 47.9 mo.

If there were at least 30 consumers and non-consumers in each of the three largest race/ethnicity groups (Hispanic, NHW, NHB), we performed exploratory analyses (i.e., we did not start with a specific hypothesis) to assess whether consumption patterns differed between race/ethnicity groups. We highlight occurrences where the difference in the percent consuming a given food between one race/ethnicity group (the comparison group) and the other two groups is large and the 95% confidence interval (95% CI) for the odds ratio (OR) for the comparison group vs. the other two groups does not contain the null value of one.

# Results

In this section, the results will be presented first in relation to the general population, followed by selected findings according to racial/ethnic differences. Findings for the percent of the general population consuming are presented for selected food groups in 3-mo intervals for infants under 6 mo, and in 6-mo intervals for infants 6 mo and older and toddlers. The interested reader may find additional results for the general population in 3-mo intervals for infants older than 6 mo and toddlers in Supplemental Table 2, and results for more food groups, and by race/ethnicity, in Supplemental Table 3 for infants (0-11.9 mo) and Supplemental Table 4 for toddlers.

## Breastfeeding and Breastfeeding Alternatives

Based on responses to the feeding practices questionnaire, which asked about practices not limited to the day of the recall, about 85% of 0- to 11.9-mo-olds were ever breastfed (Supplemental Table 1). Nearly 60% of 0- to 3.9-mo-olds were currently breastfeeding at the time of the survey, as well as almost half of 4- to 5.9- and 6- to 8.9-mo-olds and more than a third of 9- to 11.9-mo-olds (Figure 1). The prevalence of exclusive breastfeeding was much lower than that of current breastfeeding: 34% of 0- to 3.9-mo-olds and 15% of 4- to 5.9-mo-olds were exclusively breastfed, and less than 5% of 6- to 8.9-mo-olds and 9- to 11.9-mo-olds (Figure 1). Findings from the 24-h recall showed results for percent consuming breastmilk on the day of the recall very similar to percent currently breastfeeding from the feeding practices questionnaire: 58% of 0- to 3.9-mo-olds consumed breastmilk on the day of the recall, as well as 44% of 4- to 5.9- and 6- to 8.9-mo-olds, 34% of 9- to 11.9-mo-olds, and nearly a quarter of 12-14.9-mo-olds; by 21-23.9 mo, less than 5% were consuming breastmilk (Table 2).

About two-thirds of infants (less than 12 mo) consumed infant formula on the day of the survey (Table 2). Percent consuming was much lower among toddlers, with only 12% of 12-14.9-mo-olds and less than 4% of 15-23.9-mo-olds consuming formula. Less than 4% of 0-8.9-mo-olds but 17% of 9- to 11.9-mo-olds consumed cow’s milk (Table 2). Among toddlers, cow’s milk was consumed by 78-86%, with most consuming whole milk (58-71%), some consuming reduced fat milk (10-23%), and few consuming lowfat or nonfat milk (<4% except for 12-23.9-mo-olds consuming lowfat milk, 9%) (Table 2).

## Introduction of Complementary Foods (Infants 0-5.9 mo)

Seventeen percent of 0- to 3.9-mo-olds and 73% of 4- to 5.9-mo-olds consumed a complementary food on the day of the recall, and those who consumed any formula (regardless of whether they also consumed breastmilk) were more likely to consume complementary foods than those who consumed no formula (i.e., exclusively breastfed) (Table 3). Few 0- to 3.9-mo-olds who consumed no formula consumed any complementary foods (5%), but 27% of 0- to 3.9-mo-olds receiving formula consumed complementary foods. The same pattern was observed for 4- to 5.9-mo-olds: 85% of infants receiving formula consumed complementary foods compared to 51% of infants not consuming any formula. However, 4- to 5.9-mo-olds receiving formula were less likely to consume potentially non-age-appropriate complementary foods (e.g., bread rolls, pretzels, hot dogs; foods that may be less nutrient dense, contain fewer sensitive nutrients, or be a texture that is not developmentally appropriate for babies, unlike infant cereal and home-made or commercial pureed foods) compared to those consuming no formula (3.8% vs. 14%, respectively) (Table 3).

Among 4- to 5.9-mo-olds, regardless of whether they received formula, the most commonly consumed complementary food was iron-fortified infant cereal (50%); few other grains were consumed (Figure 2). Baby-food fruits (excluding 100% juice) and baby-food vegetables, respectively, were the second and third most commonly consumed complementary foods in this age group (29% and 27%). About 5% of 4- to 5.9-mo-olds consumed 100% fruit juice. Few 4- to 5.9-mo-olds consumed meats (many of which, such as beef, are iron-rich) and other non-dairy protein foods (4.3%); the most commonly consumed sources of protein were other (non-meat) foods (2.3%), while baby food meats were the least consumed (1.1%) (Table 4). However, 6.7% consumed some type of dessert, sweet, or sweetened beverage.

## Consumption Patterns of Older Infants (6-11.9 mo)

Among 6- to 11.9-mo-olds, 84% consumed a grain-based food (Table 4), with iron fortified infant cereal the most common (52%), followed by grain-based baby finger foods (33%) and family (i.e., non-infant) cereal (20%). About three quarters consumed a serving of fruit (74%) or vegetables (72%). The most commonly consumed fruits (excluding 100% fruit juice) before 12 mo were apples, bananas, and pears, and the most commonly consumed vegetables were sweet potatoes, green beans, carrots, squash, and mashed potatoes/other potato mixtures (data not shown). About 27% of 6- to 11.9-mo-olds consumed 100% fruit juice. Among 6- to 11.9-mo-olds, 41% consumed a meat or other protein, but less than 5% consumed baby-food meats. Non-baby food meats and other proteins were about equally likely to be consumed (26%). A third of 6- to 11.9-mo-olds consumed sweets, including sugar sweetened beverages (Table 4).

## Consumption Patterns of Toddlers (12-23.9 mo)

Most toddlers consumed some grain products (>90%), and about half consumed family (i.e., not infant) cereal (Table 4). For younger toddlers (12-17.9 mo), more consumed unsweetened cereal than presweetened cereal (30% vs 23%), but older toddlers (18-23.9) were equally likely to consume presweetened and unsweetened cereal (29% and 27%, respectively; Supplemental Table 4). After cereal, the most commonly consumed grain-based foods among toddlers were bread, crackers, and rice and pasta (Supplemental Table 4).

About three-quarters of toddlers consumed a fruit on the day of the survey, and a similar percentage consumed a vegetable (Table 4); however, if white potatoes are excluded, only about 60% consumed a vegetable. Most fruits consumed were fresh fruits (Supplemental Table 4). The most commonly consumed category of vegetables was white potatoes (about 33%), followed by red and orange vegetables (25%), other starchy vegetables (about 15%), dark green vegetables (about 12%), and other vegetables (<3%) (Supplemental Table 4). Nearly half of 12- to 17.9-mo-olds and just over half of 18- to 23.9-mo-olds consumed 100% fruit juice (Table 4). The AAP ([39](#_ENREF_39)) has recently recommended that children 1-3y consume no more than 4 oz (118 mL) of 100% fruit juice per day, but nearly a quarter of 12-14.9-mo-olds and nearly half of 21-23.9-mo-olds exceeded this recommendation on the day of the recall (Table 5). At the time of the survey, the AAP recommendation was no more than 6 oz (177 mL), and 14% of 12-14.9-mo-olds and 32% of 21-23.9-mo-olds exceeded that amount (Table 5).

About two-thirds of toddlers consumed meats, and about the same percentage consumed non-meat protein foods (Table 4). The most commonly consumed meat among both younger and older toddlers was chicken or turkey (39% of 12-17.9-mo-olds and 42% of 18-23.9-mo-olds), but cured meats were also consumed by more than a quarter of toddlers (Supplemental Table 4). Beef lagged farther behind at just over 10%. The most commonly consumed non-meat protein foods consumed by toddlers were cheese, eggs, yogurt, and nuts and nut butters (Supplemental Table 4).

About three-quarters of toddlers consumed sweets and SSBs (Table 4). The most commonly consumed solid sweets (i.e., not a SSB) were sweet bakery items (27% of 12-17.9-mo-olds and 36% of 18-23.9-mo-olds) and sugars, syrups, preserves, and jellies (22% of 12-17.9-mo-olds and 30% of 18-23.9-mo-olds; Supplemental Table 4). A quarter to a third of toddlers consumed a SSB on the day of the survey (27% of 12-17.9-mo-olds and 31% of 18-23.9-mo-olds; Table 4).

## Notable Differences by Race/Ethnicity

**Infants (0-11.9 mo):** Throughout the first year, NHB children were less likely than NHW and Hispanic infants to consume breastmilk on the day of the recall (43% vs. 53-59%; OR: 0.63; 95% CI: 0.36, 1.12 for ages 0- to 5.9-mo; 22% vs. 36-42%; OR: 0.43; 95% CI: 0.26, 0.71 for 6- to 11.9 mo; Table 6). NHB 0- to 3.9-mo-olds and 4- to 5.9-mo-olds also reported the lowest rates of exclusive breastfeeding (20% and 10%, respectively; Figure 1). Several notable differences by race and ethnicity were observed for specific grain products: NHB 6- to 11.9-mo-olds were much more likely than their NHW and Hispanic counterparts to consume baby-food puffs (14% vs. 27%; OR: 0.43; 95% CI: 0.24, 0.77) and ready-to-eat cereal (5% vs. 13-16%; OR: 0.33; 95% CI: 0.14, 0.77) (Table 6). Few other notable differences by race/ethnicity were observed for infants.

**Toddlers (12-23.9 mo):** Beyond the first year, NHB toddlers continue to be less likely than NHW and Hispanic toddlers to consume breastmilk (5% vs. 11-13%; OR: 0.36; 95% CI: 0.18, 0.72). In addition, NHB toddlers were more likely to consume SSBs (45% vs. 25-28%; OR: 2.31; 95% CI: 1.50, 3.57) and white potatoes (46% vs. 28-30%: OR: 2.03; 95% CI: 1.25, 3.30) than NHW and Hispanic toddlers, and less likely to consume cheese (27% vs. 34-43%: OR: 0.55; 95% CI: 0.35, 0.89). Hispanic toddlers were almost twice as likely to consume rice and pasta (35% vs. 20-25%; OR: 1.88; 95% CI: 1.06, 3.32), eggs (30% vs. 21-24%; OR: 1.7495% CI: [1.14-2.64]), and dried beans and legumes (14% vs. 8-9%; OR: 1.8495% CI: 1.06, 3.20) than non-Hispanic toddlers (Table 6) and less likely to consume sweet bakery items (22% vs. 36-37%; OR: 0.51; 95% CI: 0.31, 0.82). NHW toddlers were more likely to consume fruit than NHB and Hispanic toddlers (78% vs. 63-70%; OR: 1.72; 95% CI: 1.20, 2.48) and red and orange vegetables (26% vs. 15-20%; OR: 1.59; 95% CI: 1.08, 2.32), and less likely to consume 100% juice (37% vs. 55-56%; OR: 0.48; 95% CI: 0.35, 0.67). No notable differences were seen in the percent of toddlers consuming any meats by race and ethnicity.

# Discussion

The large sample of children under 24 mo old studied in FITS 2016 provides the best current estimates of infant and toddler food and nutrient intake and allows us to make some comparisons by race and ethnicity. Several positive findings suggest that policy and public health initiatives may be changing infant and toddler feeding practices to better align with early-feeding recommendations. However, there is some evidence that these improvements have not been consistent. To put the results of FITS 2016 in context, we have compared our results with previously published data from past FITS surveys and other relevant studies in the following discussion. The comparisons to previous FITS surveys are not a statistically rigorous trend analysis in that they do not account for changes in food group organization (which has evolved with each study iteration) or control for changes in the demographics of the US population over time.

## Breastfeeding

There appears to have been a trend for both the initiation and duration of breastfeeding to have increased in the total population over the three FITS surveys. In 2016, 83% of 4- to 23.9-mo-olds were ever breastfed, compared to 80% in FITS 2008 and 76% in 2002 ([32](#_ENREF_32)). The percentage of infants currently breastfed was also modestly higher compared to 2008 for 4- to 5.9-mo-olds (42% in 2008, 48% in 2016) and 6- to 8.9-mo-olds (37% in 2008, 46% in 2016), and remained similar for 9- to 11.9-mo-olds (37% in 2008, 36% in 2016) ([32](#_ENREF_32)). In addition, in FITS 2016, two of the Healthy People 2020 goals for breastfeeding ([38](#_ENREF_38)) have been met in the total population: percent of mothers initiating breastfeeding (Healthy People goal is 81.9%; result from FITS 2016 is 83%) and continuing to breastfeed at 12 mo (Healthy People goal is 34.1%; result from FITS 2016 is 36%). This success may be attributable to changing norms and cultural shifts in preferences to prolong nursing, as well as interventions intended to promote increased breastfeeding, such as the Baby Friendly Hospital Initiative ([42](#_ENREF_42)). For example, 21% of US births in 2016 occurred in baby friendly facilities, compared to 3% in 2007.

Despite these gains, the prevalence and duration of exclusive breastfeeding still falls short of Healthy People 2020 goals ([38](#_ENREF_38)) and AAP recommendations for exclusive breastfeeding until about 6 mo and continued breastfeeding until about 1y ([9](#_ENREF_9)). In addition, notable differences in breastfeeding behavior still exist by race and ethnicity. Although more infants overall continued to breastfeed in later infancy than in 2008, prevalence was lower among minorities. Across all age categories, prevalence of breastfeeding among NHB mothers was lower than prevalence of breastfeeding among the total population. Prevalence of exclusive breastfeeding up to 6 mo was appreciably lower for NHB infants compared to NHW and Hispanic infants. As such, the progress in overall breastfeeding initiation and moderate success in extending breastfeeding duration masks important racial/ethnic differences and emphasizes the need to address these disparities. Previous research has shown that approximately 60% of women stop breastfeeding earlier than they would like ([43](#_ENREF_43)). Known barriers to breastfeeding include lack of family, peer, and health provider support; lack of social/cultural acceptance; inadequate knowledge; preference to bottle feed; difficulties around initiation (e.g., latching problems, breast discomfort); and the need to return to work ([24](#_ENREF_24), [44](#_ENREF_44)). Mothers who returned to work within six weeks postpartum were three times as likely to stop breastfeeding before the recommended 6 mo, after adjusting for race, income, and education ([45](#_ENREF_45)). Inequities in paid parental leave may further contribute to the breastfeeding disparity seen among low-income and racial minority groups ([46](#_ENREF_46)).

## Complementary Foods

FITS 2016 also provides evidence of success in complementary feeding practices, such as timing of introduction of complementary foods, while signaling the need for improvement in others, such as low consumption of iron-rich foods, fruits, and vegetables, and excess consumption of 100% juice and SSBs.

**Age of introduction of complementary foods:** Similar to recent findings from NHANES 2009–2012 ([47](#_ENREF_47)), the prevalence of early introduction of complementary foods continues to decline among the general population, with only 17% infants under 4 mo being introduced to foods other than breastmilk or formula in 2016. However, introduction of complementary foods before 4 mo was considerably higher among infants who consumed any formula compared to those who consumed no formula. The age of introduction of complementary foods and its association with overweight and obesity in later childhood remains equivocal, with only a few studies showing that early introduction before 4 months of age may be associated with a higher body mass index, while the majority of studies failed to show a relationship between age of introduction of complementary foods and later adiposity in childhood ([5](#_ENREF_5), [48](#_ENREF_48), [49](#_ENREF_49)).

**Consumption of iron-rich foods:** Sixteen percent of 1- to 2-year-olds were reported in 2010 to be iron-deficient ([50](#_ENREF_50)), and evidence from longitudinal studies highlight some of the long-term cognitive impairments associated with iron deficiency in early childhood ([51](#_ENREF_51)). Iron deficiency is caused by multiple factors, including insufficient intake, genetic factors affecting iron metabolism, and excessive losses. Both globally and in the US, iron deficiency is common in young children ([52](#_ENREF_52)). The AAP recommends that full-term infants who are partially or exclusively breastfed receive about 1 mg of iron/kg/day starting at 4 to 6 mo, in the form of a liquid iron supplement, until iron-rich foods like iron-fortified infant cereal and iron-rich meats are introduced ([50](#_ENREF_50)). In FITS 2008, 12% of 6- to 11.9-mo-olds consumed less than the Estimated Average Requirement for iron, an increase from 7% in FITS 2002 ([53](#_ENREF_53)). Elsewhere in this supplement, FITS 2016 data show that figure has increased to 18% ([52](#_ENREF_52)). In addition, among 6- to 11.9-mo-olds, only 15% consumed any type of supplement and <5% consumed iron supplements specifically ([52](#_ENREF_52)). Iron-fortified infant cereal is often thought of as the first solid food introduced to infants. However, consumption of iron-fortified infant cereal declined since 2008—55% of 6- to 8.9-mo-olds in 2016 (Supplemental Table 2) vs 82% and 79% in 2002 and 2008, respectively ([32](#_ENREF_32)). This decline is not compensated for by the consumption of iron-rich pureed baby meats, which is also low and peaked at 5% among 6- to 8.9-mo-olds (Supplemental Table 2).

Another compounding contributor to iron-deficiency in this age group is the early introduction and consumption of cow’s milk, which has been associated with gastrointestinal blood loss in infants from conditions like food protein–induced allergic proctocolitis and cow’s milk protein allergy ([54](#_ENREF_54), [55](#_ENREF_55)). The AAP recommends that parents avoid the introduction of cow’s milk before 12 mo ([56](#_ENREF_56)). The prevalence of cow’s milk consumption earlier than 12 mo remained at about 17% of 9- to 11.9-mo-olds compared to FITS 2008 ([32](#_ENREF_32)); however, those infants consuming cow’s milk tended to consume large quantities. These observations highlight the need for continued education of caregivers about the avoidance of cow’s milk in the first year of life and the need for efforts to educate parents and caregivers of the sources and importance of iron in their child’s diet.

**Fruits, 100% fruit juice, and vegetables:** Fruit and vegetable consumption for infants under 9 mo continued to increase or remain level. In FITS 2016, 70% of 6- to 8.9-mo-olds and 79% of 9- to 11.9-mo-olds consumed any fruit (Supplemental Table 2) compared to 65% and 81% in FITS 2008 ([32](#_ENREF_32)), and 76% of 6- to 8.9-mo-olds and 78% of 9- to 11.9-mo-olds consumed any vegetable (Supplemental Table 2) compared to 63% and 72% in FITS 2008 ([32](#_ENREF_32)). For toddlers, about 80% consumed a fruit (down somewhat from 85-90% in 2008 ([32](#_ENREF_32))) and about 70% consumed a vegetable (about the same as 2008 ([32](#_ENREF_32))). Nonetheless, as with recent findings in NHANES ([47](#_ENREF_47)) and FITS 2008 ([32](#_ENREF_32)), the amount and variety of fruit and vegetable consumption remained far below recommendations in many older infants, and fall even shorter in toddlers. The AAP recommends offering a fruit or vegetable at every meal and snack after 6 mo ([57](#_ENREF_57)). However, more than 20% of 12- to 23.9-mo-olds did not consume a single serving of fruit on the day of recall, and 30% did not consume any vegetable servings.

Several areas for improvement were noted regarding types of fruits and vegetables consumed among 12- to 23.9-mo-olds. Although toddlers were about as likely to consume a serving of vegetable (71-74%, Supplemental Table 2) as a serving of fruit (71-81%), only about 60% consumed a vegetable other than white potatoes, and about a third consumed white potatoes.

Recent studies have linked consumption of fruits and vegetables to a decreased risk of several chronic diseases and obesity ([15](#_ENREF_15), [58-69](#_ENREF_58)), and early feeding preferences may persist throughout the lifespan ([70](#_ENREF_70)). Innovative approaches to introduce and sustain the consumption of a variety of fruits and vegetables should be a high priority, not just for infant feeding but also for families.

In 2017, the AAP updated its position on fruit juice and recommended delaying the introduction of juice until 12 mo and limiting the maximum amount of juice for young toddlers to no more than 4 oz (118 mL) of 100% fruit juice per day ([58](#_ENREF_58), [71](#_ENREF_71), [72](#_ENREF_72)). However, at the time of the survey, the previous recommendation from the AAP allowed for no more than 4-6 oz (118–177 mL) daily starting in late infancy. A recent meta-analysis concluded that consumption of 100% juice was associated with a small amount of excess weight gain in childhood ([58](#_ENREF_58)). We found that infants and toddlers were somewhat less likely to consume 100% juice in 2016 than in FITS 2008 ([32](#_ENREF_32)), with the greatest decrease between 6 and 12 mo (e.g., for 6- to 11.9-mo-olds, percent consuming 100% juice fell from 31-41% in 2008 ([32](#_ENREF_32)) to 22-33% in 2016; Supplemental Table 2). However, a third of 9- to 11.9-mo-olds still drank 100% juice (Supplemental Table 2), which is not consistent with the most recent AAP recommendations to delay the introduction of fruit juice until 12 mo ([39](#_ENREF_39)). Further, nearly one quarter of the youngest toddlers (12-14.9 mo) were consuming more than the new recommended maximum amount of 4 oz (118 mL) of 100% fruit juice per day, and 14% were consuming more than the previously recommended maximum amount of 6 oz (177 mL) ([40](#_ENREF_40)). Nearly half of toddlers 21-23.9 mo were consuming more than 4 oz (118 mL) and nearly a third were consuming more than 6 oz (177 mL) per day.

**Sugar-sweetened beverages:** Consumption of SSBs has risen for some age groups and fallen for others since 2008. In 2016, few younger infants (under 6 mo) consumed SSBs, but among 9- to 11.9-mo-olds, the percent consuming rose from 11% in 2008 ([32](#_ENREF_32)) to 14% in 2016 (Supplemental Table 2) and doubled—from 14% in 2008 ([32](#_ENREF_32)) to 28% in 2016 (Supplemental Table 2)—for 12- to 14.9-mo-olds. Peak consumption in 2016 was in 18- to 20.9-mo-olds (33% consuming an average of 106 kcal per consumer (Supplemental Table 2). Clearly, there is opportunity to further delay the introduction of SSBs and to reduce consumption in late infancy and toddlerhood. There were racial/ethnic disparities in consumption of SSBs: NHB toddlers were more likely to consume SSBs than NHW and Hispanic toddlers. Sugary drinks should be avoided because they contribute calories but few nutrients and may decrease the child’s appetite for more nutritious foods. Moreover, the consumption of high caloric density foods and beverages in early childhood significantly increases the likelihood of consumption of these products later in childhood, increases the risk for dental caries, and may contribute to the prevalence of early childhood obesity ([15](#_ENREF_15), [60](#_ENREF_60), [62](#_ENREF_62), [63](#_ENREF_63)).

## Study Strengths and Limitations

Data from the FITS 2016 can make an important contribution to early-childhood nutrition. The study provides population level data, and builds on previous FITS studies, which in turns can help us to monitor the diets of very young children in the US. It can allow us to compare feeding practices to national recommendations and also contribute to new dietary guidelines for population aged from 0-2y.

A potential limitation of the FITS 2016 study applies to all cross-sectional studies that use self-reported 24-h recall data, because they are subject to reporting bias and errors in remembering what and how much was consumed. Some of the race/ethnicity differences in food consumption patterns that we found may be due to household economic and education status, access to food (related to geographic location, i.e., food deserts), and other social determinants rather than, or in addition to, race/ethnicity. Previous research has yielded conflicting conclusions about the role of race/ethnicity on breastfeeding. One study found that maternal education had a greater effect on breastfeeding incidence than race/ethnicity ([73](#_ENREF_73)), while another reported that even after accounting for socioeconomic status NHB were consistently less likely to breastfeed compared to NHW ([74](#_ENREF_74), [75](#_ENREF_75)). The analysis of differences between racial/ethnic groups was exploratory, as previous FITS studies did not present results by race/ethnicity, and we did not have any specific hypotheses we were testing. However, the race/ethnicity differences we report are largely consistent with the limited data available in the literature ([47](#_ENREF_47)) and suggest that while national data from the general population are useful for documenting trends, it is critical that they take into account race/ethnicity during study design, data collection, and analysis. Future analyses are planned and include examining racial/ethnic differences in FITS 2002 and 2008 surveys (informed by the results reported here) and conducting in-depth trend analyses.

## Opportunities for Future Research

The most recent data from the FITS appear to indicate that we have found some improvements in breastfeeding practices and early introduction of complementary foods; however, several areas of concern remain. These include exclusive breastfeeding; timing of introduction of complementary foods, particularly in formula-fed infants; low consumption of iron-rich foods; early introduction of cow’s milk; inadequate quantity and variety of fruits and vegetables consumed; and early introduction and consumption of SSBs and 100% juice in the first year of life. In addition, the racial/ethnic disparities in feeding practices highlighted herein may be heralds of disparities in obesity and metabolic disorders later in life ([22](#_ENREF_22), [24](#_ENREF_24), [76-78](#_ENREF_76)).

Factors that account for the departure from the feeding practices recommended by the AAP remain uncertain. The design of FITS 2016 and the substantial data collection necessary to describe patterns of food consumption limits somewhat our ability to measure and understand the underlying attitudes, beliefs, socioeconomic, and potential environmental factors that led to these patterns. Associated lifestyle factors that may influence consumption patterns will be explored in subsequent publications. Although dietary counseling by primary care providers is a part of routine care, the quality and effectiveness of such counseling may need to be strengthened to be inclusive of cultural preferences, practices, and access to nutritious and affordable foods. To our knowledge, the frequency, quality, and efficacy with which counseling routinely addresses practices like avoiding the early introduction of solids or cow’s milk has not been carefully examined ([79](#_ENREF_79)). Furthermore, we are not aware of any studies that have effectively addressed the substantial intake of high caloric density foods that may lead to the development of early obesity. FITS 2016 offers many opportunities to fill further data gaps in relation to the feeding of infants and toddlers. Future exploration of the meal, snack, and beverage patterns in this age group is planned, and hopefully will shed light on potential obesogenic dietary patterns. In addition, examining food sources of nutrients could provide more insights into how foods selected by caregivers contribute to nutrient intakes, and allow for exploration of differences by race and ethnicity, WIC status, or socioeconomic status. Ongoing surveillance of early childhood food and beverage consumption that accounts for race and ethnicity is needed to identify feeding patterns that may lead to future health problems early enough to intervene and change behaviors.

Across race and ethnicity, our results are largely consistent with the limited data available in the literature ([47](#_ENREF_47)) and underline the need for focused education and policy changes to decrease some of the disparities observed in feeding practices and dietary intakes. Whether the differences in food consumption patterns reflect cultural preferences, choices directed by access to food or food insecurity, or other social justice issues remains uncertain. These differences could also be due to household economic and education status rather than race and ethnicity. Our observations point to the need to tailor education and feeding guidance to specific racial and ethnic groups.

## Conclusion

FITS 2016 provides the most recent, cross-sectional, estimates of food and beverage consumption for young children in the United States. The sample size of FITS permits exploration of racial/ ethnic comparisons that have not previously been described, and while initial findings regarding differences by race/ethnicity herein are exploratory, our data may set the stage for further research and provide important insights into guidance and interventions that are still needed in order to improve diets.

# Author Contributions

A.S.A, D.J.C., E.F.J. designed research; D.J.C. performed statistical analysis; A.A.R., W.H.D., A.C.L, R.C., A.S.A., and E.F.J. analyzed or interpreted data and wrote the paper. A.A.R. had primary responsibility for final content. All authors read and approved the final manuscript.

# Acknowledgements

The study upon which the data presented here are based was a collaborative effort of many organizations and individuals; those are listed in the acknowledgements to the companion Methods and Study Design paper ([33](#_ENREF_33)). In addition, we wish to acknowledge Prof. Johanna Dwyer (Tufts University and the National Institutes of Health, Office of Dietary Supplements) and Virginia A. Stallings (Children’s Hospital of Philadelphia) for their guidance on current issues in evaluating food consumption.

# References

1. Ogden C, Carroll M, Kit B, Flegal K. Prevalence of childhood and adult obesity in the United States, 2011-2012. JAMA. 2014;311(8):806-14.

2. Ogden C, Carroll M, Lawman H, Fryar C, Kruszon-Moran D, Kit B, Flegal K. Trends in obesity prevalence among children and adolescents in the United States, 1988-1994 Through 2013-2014. JAMA. 2016;315(21):2292-9

3. Cunningham S, Kramer M, Narayan K. Incidence of childhood obesity in the United States. N Engl J Med. 2014;370(5):403-11.

4. Horta B, Loret de Mola C, Victora C. Long‐term consequences of breastfeeding on cholesterol, obesity, systolic blood pressure and type 2 diabetes: a systematic review and meta‐analysis. Acta Paediatr 2015;104(S467):30-7.

5. Patro-Gołąb B, Zalewski M, Kołodziej M, Kouwenhoven S, Poston L, Godfrey K, Koletzko B, van Goudoever J, Szajewska H. Nutritional interventions or exposures in infants and children aged up to 3 years and their effects on subsequent risk of overweight, obesity and body fat: a systematic review of systematic reviews. Obes Rev 2016;17(12):1245-57.

6. Pan L, Freedman D, Sharma A, Castellanos-Brown K, Park S, Smith R, Blanck H. Trends in obesity among participants aged 2-4 years in the Special Supplemental Nutrition Program for Women, Infants, and Children—United States, 2000-2. MMWR. 2016;65(45):1256–60.

7. Isong IA, Rao SR, Bind M-A, Avendaño M, Kawachi I, Richmond TK. Racial and ethnic disparities in early childhood obesity Pediatrics. 2018;141(1):e20170865. doi: 10.1542/peds.2017-0865.

8. Thompson A, Bentley M. The critical period of infant feeding for the development of early disparities in obesity. Soc Sci Med. 2013;97:288-96. doi: 10.1016/j.socscimed.2012.12.007.

9. American Academy of Pediatrics (Section on Breastfeeding). Policy Statement: Breastfeeding and the use of human milk. Pediatrics 2012;129(3):e827–41.

10. Rendall M, Weden M, Fernandes M, Vaynman I. Hispanic and black US children's paths to high adolescent obesity prevalence. Pediatr Obes. 2012;7:423-35.

11. Skinner A, Skelton J. Prevalence and trends in obesity and severe obesity among children in the United States, 1999-2012. JAMA Pediatr. 2014;168(6):561-6.

12. Wang Y, Beydoun M. The obesity epidemic in the United States--gender, age, socioeconomic, racial/ethnic, and geographic characteristics: a systematic review and meta-regression analysis. Epidemiol Rev. 2007;29:6-28.

13. Dewey K, Heinig M, Nommsen-Rivers L. Differences in morbidity between breast-fed and formula-fed infants. J Pediatr 1995;126:696–70.

14. Gartner L, Morton J, Lawrence R, Naylor A, O'Hare D, Schanler R, Eidelman A. Breastfeeding and the use of human milk. Pediatrics. 2005;115(2):496-506.

15. Pan L, Li R, Park S, Galuska D, Sherry B, Freedman D. A longitudinal analysis of sugar-sweetened beverage intake in infancy and obesity at 6 years. Pediatrics. 2014;134(Suppl 1):S29-35.

16. Slusser W. Breastfeeding and maternal and infant health outcomes in developed countries. AAP Grand Rounds 2007;18(2):15-6.

17. Hauck F, Thompson J, Tanabe K, Moon R, Vennemann M. Breastfeeding and reduced risk of Sudden Infant Death Syndrome: a meta-analysis. Pediatrics 2011;128:103–10.

18. Li R, Dee D, Li C-M, Hoffman H, Grummer-Strawn L. Breastfeeding and risk of infections at 6 years. Pediatrics. 2014;134(Suppl 1):S13-20.

19. Moorman J, Zahran H, Truman B, Molla M. Current asthma prevalence—United States, 2006–2008. MMWR. 2011;60(Suppl):84–6.

20. Kitsantas P, Gaffney K. Racial/ethnic disparities in infant mortality. J Perinat Med 2010;38:87–94.

21. Freedman D. Obesity–United States, 1988-2008. MMWR. 2011;60(Jan 14 Suppl):73-7.

22. Bartick M, Jegier B, Green B, Schwarz E, Reinhold A, Stuebe A. Disparities in breastfeeding: impact on maternal and child health outcomes and costs. J Pediatr 2017;181:49-55. doi: doi: 10.1016/j.jpeds.2016.10.028.

23. Centers for Disease Control and Prevention. Breastfeeding Report Card: Progressing Toward National Breastfeeding Goals, United States 2016. Atlanta: CDC; 2016.

24. Jones K, Power M, Queenan J, Schulkin J. Racial and ethnic disparities in breastfeeding. Breastfeed Med 2015;10:186–96.

25. Sparks P. Racial/ethnic differences in breastfeeding duration among WIC-eligible families. Womens Health Issues 2011;21:374–82.

26. Holt K, Wooldridge N, Story M, Sofka D. Bright Futures Nutrition. 3rd ed: The American Academy of Pediatrics; 2011.

27. American Academy of Pediatrics (Committee on Nutrition). Chapter 6: Complementary Feeding. In: Kleinman R, editor. Pediatric Nutrition Handbook. 6th ed. Elk Grove Village, IL: American Academy of Pediatrics; 2009.

28. World Health Organization. Global strategy for infant and young child feeding. Geneva: 2003.

29. Kuo A, Inkelas M, Slusser W, Maidenberg M, Halfon N. Introduction of solid food to young infants. Matern Child Health J. 2011;15(8):1185–94.

30. Fein S, Labiner-Wolfe J, Shealy K, Li R, Chen J, Grummer-Strawn L. Infant Feeding Practices Study II: study methods. Pediatrics. 2008;122(Suppl 2):S28-35.

31. Raiten D, Raghavan R, Porter A, Obbagy J, Spahn J. Executive summary: Evaluating the evidence base to support the inclusion of infants and children from birth to 24 mo of age in the Dietary Guidelines for Americans--"the B-24 Project". Am J Clin Nutr 2014;99:663S-91S.

32. Siega-Riz AM, Deming DM, Reidy KC, Fox MK, Condon E, Briefel RR. Food consumption patterns of infants and toddlers: where are we now? J Am Diet Assoc. 2010;110(12):S38-S51. doi: 10.1016/j.jada.2010.09.001.

33. Anater AS, Catellier DJ, Levine BA, Krotki KP, Jacquier EF, Eldridge AL, Bronstein KE, Harnack LJ, Lorenzana Peasley JM, Lutes AC. The Feeding Infants and Toddlers Study (FITS) 2016: Study Design and Methods. J Nutr. 2018.

34. Devaney B, Kalb L, Briefel R, Zavitsky-Novak T, Clusen N, Ziegler P. Feeding Infants and Toddlers Study: overview of the study design. J Am Diet Assoc. 2004;104:8-13. doi: 10.1016/j.jada.2003.10.023.

35. Briefel RR, Kalb LM, Condon E, Deming DM, Clusen NA, Fox MK, Harnack L, Gemmill E, Stevens M, Reidy KC. The Feeding Infants and Toddlers Study 2008: study design and methods. J Am Diet Assoc. 2010;110(12 Suppl):S16-26. doi: 10.1016/j.jada.2010.09.005.

36. Rhodes D, Adler M, Clemens J, Moshfegh A. What we eat in America food categories and changes between survey cycles. J Food Comp Anal. 2017;64:107-11.

37. Institute of Medicine (Food and Nutrition Board). Dietary Reference Intakes: Applications in Dietary Assessment. Washington, DC: National Academies Press; 2000.

38. US Department of Health and Human Services. Healthy People 2020: Maternal, Infant, and Child Health Objectives MICH-21.1 through 21.5: Office of Disease Prevention and Health Promotion; 2010 [updated 2012; cited 2018 March 26]. Available from: <https://www.healthypeople.gov/2020/topics-objectives/topic/Maternal-Infant-and-Child-Health/objectives>.

39. American Academy of Pediatrics. Where We Stand: Fruit Juice 2017 [updated May 22, 2017; cited 2017 June 1]. Available from: <https://www.healthychildren.org/English/healthy-living/nutrition/Pages/Where-We-Stand-Fruit-Juice.aspx>.

40. American Academy of Pediatrics (Committee on Nutrition). The use and misuse of fruit juice in pediatrics. Pediatrics. 2001;107(5):1210-3. doi: 10.1542/peds.107.5.1210.

41. American Academy of Pediatrics. AAP publications retired or reaffirmed, October 2006. Pediatrics. 2007;119(2):405.

42. Munn A, Newman S, Mueller M, Phillips S, Taylor S. The impact in the United States of the baby-friendly hospital initiative on early infant health and breastfeeding outcomes. Breastfeed Med. 2016;11(5):222-30.

43. Odom E, Li R, Scanlon K, Perrine C, Grummer-Strawn L. Reasons for earlier than desired cessation of breastfeeding. Pediatrics. 2013;131(3):e726-32.

44. Gross TT, Powell R, Anderson AK, Hall J, Davis M, Hilyard K. WIC peer counselors' perceptions of breastfeeding in African American women with lower incomes. J Hum Lact. 2015;31(1):99-110. doi: <https://doi.org/10.1177/0890334414561061>.

45. Guendelman S, Kosa J, Pearl M, Graham S, Goodman J, Kharrazi M. Juggling work and Breastfeeding: effects of maternity leave and occupational characteristics. Pediatrics 2009;123(1):e38-e46.

46. Ogbuanu C, Glover S, Probst J, Liu J, Hussey J. The effect of maternity leave length and time of return to work on breastfeeding. Pediatrics. 2011;127(6):e1414-27.

47. Miles G, Siega-Riz AM. Trends in food and beverage consumption among infants and toddlers: 2005-2012. Pediatrics. 2017. Epub May 1, 2017. doi: 10.1542/peds.2016-3290.

48. Burdette HL, Whitaker RC, Hall WC, Daniels SR. Breastfeeding, introduction of complementary foods, and adiposity at 5 y of age. Am J Clin Nutr 2006;83(3):550-8.

49. Pierce J, Taylor M, Langley-Evans S. Timing of the introduction of complementary feeding and risk of childhood obesity: a systematic review Int J Obes 2013;37:1295-306.

50. Baker R, Greer F. Diagnosis and prevention of iron deficiency and iron-deficiency anemia in infants and young children (0-3 years of age). Pediatrics. 2010;126(5):1040-50.

51. Lozoff B, Castillo M, Clark K, Smith J, Sturza J. Iron Supplementation in Infancy Contributes to More Adaptive Behavior at 10 Years of Age. J Nutr. 2014;144(6):838-45.

52. Bailey RL, Catellier DJ, Jun S, Dwyer J, Jacquier EF, Anater AS, Eldridge AL. Total Usual Nutrient Intakes of U.S. Children (<48 mo): Findings from the Feeding Infants and Toddlers Study (FITS) 2016. J Nutr. 2018.

53. Butte NF, Fox MK, Briefel RR, Siega-Riz AM, Dwyer JT, Deming DM, Reidy KC. Nutrient intakes of US infants, toddlers, and preschoolers meet or exceed Dietary Reference Intakes. J Am Diet Assoc. 2010;110(12):S27-S37. doi: 10.1016/j.jada.2010.09.004.

54. Nowak-Węgrzyn A, Katz Y, Mehr S, Koletzko S. Non-IgE-mediated gastrointestinal food allergy. J Allergy Clin Immunol. 2015;135(5):1114-24.

55. Ziegler E. Consumption of cow's milk as a cause of iron deficiency in infants and todd. Nutr Rev. 2011;69(s1).

56. American Academy of Pediatrics (Committee on Nutrition). The use of whole cow's milk in infancy. Pediatrics. 1992;89(6):1105-9.

57. American Academy of Pediatrics. About HALF: Healthy Active Living for Families (HALF) Program 2012 [updated not specified; cited 2018 April 3]. Available from: <https://www.aap.org/en-us/advocacy-and-policy/aap-health-initiatives/HALF-Implementation-Guide/Pages/About-HALF.aspx>.

58. Auerbach B, Wolf F, Hikida A, Vallila-Buchman P, Littman A, Thompson D, Louden D, Taber D, Krieger J. Fruit juice and change in BMI: A meta-analysis. Pediatrics. 2017 139(4):e20162454.

59. Dietz W. Childhood weight affects adult morbidity and mortality. J Nutr. 1998;128(2):411-4S.

60. Ebbeling C, Feldman H, Chomitz V, Antonelli TA, Gortmaker SL, Osganian SK, Ludwig DS. A randomized trial of sugar-sweetened beverages and adolescent body weight. N Engl J Med. 2012;367(15):1407–16.

61. Institute of Medicine. Early Childhood Obesity Prevention Policies: Consensus Report. 2011.

62. Ludwig D, Peterson K, Gortmaker S. Relation between consumption of sugar sweetened drinks and childhood obesity: a prospective, observational analysis. Lancet. 2001;357(9255):505-8.

63. Park S, Pan L, Sherry B, Li R. The association of sugar-sweetened beverage intake during infancy with sugar-sweetened beverage intake at 6 years of age. Pediatrics. 2014;134(S1):S56-62.

64. Pocock M, Trivedi D, Wills W, Bunn F, Magnusson J. Parental perceptions regarding healthy behaviours for preventing overweight and obesity in young children: A systematic review of qualitative studies. Obesity Review. 2010;11(5):338-53.

65. He F, Nowson C, Lucas M, MacGregor G. Increased consumption of fruit and vegetables is related to a reduced risk of coronary heart disease: meta-analysis of cohort studies. J Hum Hypertens. 2007 21(9):717-28.

66. He F, Nowson C, MacGregor G. Fruit and vegetable consumption and stroke: meta-analysis of cohort studies. Lancet. 2006;367(9507):320-6.

67. Hodder R, Stacey F, O'Brien K, Wyse R, Clinton-McHarg T, Tzelepis F, James E, Bartlem K, Nathan N, Sutherland R, et al. Interventions for increasing fruit and vegetable consumption in children aged five years and under. Cochrane Database Syst Rev. 2018;1:CD008552. Epub Jan 25, 2018. doi: 10.1002/14651858.CD008552.pub4.

68. Wu L, Sun D, He Y. Fruit and vegetables consumption and incident hypertension: dose-response meta-analysis of prospective cohort studies. J Hum Hypertens. 2016;30(10):573-80. doi: 10.1038/jhh.2016.44.

69. Zhan J, Liu Y, Cai L, Xu F, Xie T, He Q. Fruit and vegetable consumption and risk of cardiovascular disease: A meta-analysis of prospective cohort studies. Crit Rev Food Sci Nutr. 2017;57(8):1650-63. doi: 10.1080/10408398.2015.1008980

70. Birch L, Savage J, Ventura A. Influences on the development of children's eating behaviours: from infancy to adolescence. Can J Diet Pract Res 2007 68(1):s1.

71. Abrams S, Daniels S. Fruit juice and child health. Pediatrics. 2017;139(4):e20170041. doi: 10.1542/peds.2017-0041.

72. Heyman M, Abrams S. Fruit juice in infants, children, and adolescents: current recommendations. Pediatrics. 2017;139(6):e20170967.

73. Kurinij N, Shiono P, Rhoads G. Breastfeeding incidence and duration in black and white women. Pediatrics 1988;81:365-71.

74. Li R, Grummer-Strawn L. Racial and ethnic disparities in breastfeeding among United States infants: third National Health and Nutrition Examination Survey, 1988–1994. Birth 2002 29(4):251-7.

75. Rassin D, Richardson C, Baranowski T, Nader PR, Guenther N, Bee DE, Brown JP. Incidence of breastfeeding in a low socioeconomic group of mothers in the United States: ethnic patterns. Pediatrics 1984;73(2):132-7.

76. American Cancer Society. Breast Cancer Facts & Figures, 2009-2010. Atlanta, GA2010.

77. Arenz S, Rückerl R, Koletzko B, von Kries R. Breast-feeding and childhood obesity—a systematic review. Int J Obes. 2004 28(10):1247-56.

78. Fejerman L, Stern M, John E, Torres-Mejía G, Hines L, Wolff R, Baumgartner K, Giuliano A, Ziv E, Pérez-Stable E, et al. Interaction between common breast cancer susceptibility variants, genetic ancestry, and nongenetic risk factors in Hispanic women. Cancer Epidemiol Biomarkers Prev 2015;24(11):1731-9.

79. Daniels S, Hassink S. The role of the pediatrician in primary prevention of obesity. Pediatrics. 2015;136(1):e275-92. doi: doi: 10.1542/peds.2015-1558.

# Figure Titles

Figure 1. Current and exclusive breastfeeding rates by race and ethnicity, from FITS 2016 feeding practices questionnaire. Lines represent Healthy People 2020 goals ([38](#_ENREF_38)) for exclusive breastfeeding at 3 mo (0-3.9 mo columns) and 6 mo (4-5.9 mo columns, solid line) and for any breastfeeding at 6 mo (4-5.9 mo columns, dashed line) and 12 mo (9-11.9 mo columns). No Healthy People 2020 goals are set for breastfeeding at 9 mo. See Table 1 for sample sizes. [original to this manuscript]

Figure 2. Percentage of infants consuming complementary foods by age, from FITS 2016 24h dietary recall. * may include other grains; ** excludes cheese and yogurt; † excludes fruit juice; †† includes sugar sweetened beverages. See Table 1 for sample sizes. [original to this manuscript]

# Tables

Table 1. Sample sizes by race/ethnicity for infants 0-11.9 mo^1^

| Child’s Age | Sample size, *n* | | | |
| --- | --- | --- | --- | --- |
|  | Overall | NHW | Hispanic | NHB |
| 0-3.9 mo | 305 | 198 | 49 | 38 |
| 4-5.9 mo | 294 | 191 | 47 | 38 |
| 6-8.9 mo | 465 | 318 | 62 | 62 |
| 9-11.9 mo | 430 | 294 | 63 | 51 |

^1^ Values are sample sizes corresponding to data presented in Figures 1 (all columns) and 2 (overall column).

Table 2. Consumption of breastmilk, infant formula, and cow’s milk during a single 24-h dietary recall, general population, children 0-23.9 mo^1^

| Food Group | Child's Age, mo | | | | | | | |
| --- | --- | --- | --- | --- | --- | --- | --- | --- |
|  | 0-3.9  (*n*=305) | 4-5.9  (*n*=295) | 6-8.9  (*n*=468) | 9-11.9  (*n*=434) | 12-14.9  (*n*=412) | 15-17.9  (*n*=308) | 18-20.9  (*n*=251) | 21-23.9  (*n*=162) |
| Breastmilk | 58 | 44 | 44 | 34 | 24 | 12 | 5.5 | 4.7 |
| Infant formula | 59 | 69 | 67 | 63 | 12 | 3.1 | 1.6 | 0.5 |
| Any cow's milk^2^ | 1.8 | 2.0 | 3.7 | 17 | 78 | 84 | 83 | 86 |
| Whole | 1.4 | 1.2 | 1.5 | 10 | 67 | 71 | 71 | 58 |
| Reduced fat | 0 | 0.7 | 1.4 | 5.6 | 11 | 10 | 13 | 23 |
| Lowfat | 0.4 | 0 | 0.8 | 1.5 | 3.2 | 3.3 | 3.5 | 9.0 |
| Nonfat | 0 | 0.2 | 0 | 0.2 | 1.7 | 2.4 | 3.6 | 2.5 |

^1^ Values are mean percentage of children consuming the food category during a single 24-h recall.

^2^ Includes all fat levels, as well as flavored, unflavored, or powdered.

Table 3. Consumption of complementary foods during a single dietary recall by breastfeeding status, children 0 to 5.9 mo^1^

| Type of  Complementary Foods | Child’s Age, mo | | | | | | |
| --- | --- | --- | --- | --- | --- | --- | --- |
|  | 0-3.9 | | |  | 4-5.9 | | |
|  | Overall  (*n*=305) | Consumed no formula  (*n*=139) | Consumed any formula^2^  (*n*=162) |  | Overall  (*n*=295) | Consumed no formula  (*n*=100) | Consumed any formula^2^  (*n*=192) |
| No complementary foods | 83 | 95 | 73 |  | 27 | 49 | 15 |
| Any complementary foods | 17 | 5 | 27 |  | 73 | 51 | 85 |
| Infant cereal only^3^ | 5.7 | 2.3 | 8.7 |  | 13 | 5.9 | 17 |
| Pureed baby foods^4^ | 6.4 | 0.8 | 11 |  | 53 | 31 | 64 |
| Other food or beverage^5^ | 4.6 | 1.8 | 6.9 |  | 7.3 | 14 | 3.8 |

^1^ Values are mean percentage of children consuming the complementary food category during a single 24-h recall.

^2^ Includes children who consumed both formula and breastmilk as well as children who consumed formula and no breastmilk.

^3^ Includes any kind of infant cereal, regardless of grain (i.e., rice, oat, quinoa, wheat, multigrain, or unknown grain).

^4^ Includes any pureed fruit, vegetable, or meat, whether commercial jarred baby-food or homemade pureed fresh foods; child may also consume infant cereal in addition to these but does not consume other (not pureed) foods.

^5^ Includes any food that is not infant cereal or commercial or homemade pureed baby food.

Table 4. Consumption of complementary foods during a single 24-h dietary recall, general population, children 0-23.9 mo^1^

| Food Group | Child's Age, mo | | | | |
| --- | --- | --- | --- | --- | --- |
|  | 0-3.9  (*n*=305) | 4-5.9  (*n*=295) | 6-11.9  (*n*=902) | 12-17.9  (*n*=720) | 18-23.9  (*n*=413) |
| Any grain products | 14 | 54 | 84 | 94 | 96 |
| Infant cereal^2^ | 13 | 50 | 52 | 16 | 5.3 |
| Family cereal^3^ | 0.6 | 1.1 | 20 | 50 | 58 |
| Baby finger foods^4^ | 1.1 | 4.4 | 33 | 16 | 5.7 |
| Any fruit^5^ | 6.5 | 37 | 74 | 75 | 79 |
| Any 100% juice^6^ | 4.2 | 5.5 | 27 | 45 | 55 |
| Any vegetable^7^ | 2.9 | 34 | 72 | 73 | 71 |
| Baby-food vegetables^8^ | 1.5 | 27 | 45 | 8.7 | 2.5 |
| Vegetables (not baby food)^9^ | 0.9 | 7.6 | 29 | 58 | 60 |
| White potatoes^10^ | 0.7 | 2.7 | 15 | 33 | 32 |
| Any meat/other protein food^11^ | 1.7 | 4.3 | 41 | 88 | 91 |
| Baby-food meats | 0.2 | 1.1 | 4.2 | 0.9 | 0.6 |
| Meats (not baby food) | 1.1 | 1.3 | 26 | 68 | 72 |
| Other protein sources^12^ | 0.8 | 2.3 | 26 | 68 | 75 |
| Any sweets/sugar-sweetened beverage^13^ | 1.4 | 6.7 | 34 | 73 | 80 |
| Sugar sweetened beverages | 0.1 | 2.3 | 8.5 | 27 | 31 |
| Savory snacks^14^ | 0.4 | 0.7 | 77 | 17 | 20 |

^1^ Values are mean percentage of children consuming the food category during a single 24-h recall.

^2^ Includes any kind of baby-food cereal, regardless of grain (i.e., rice, oat, quinoa, wheat, multigrain, or unknown grain)

^3^ Includes any ready-to-eat or hot cereal that is not infant cereal.

^4^ Includes pretzels, crackers, rice cakes, and baby-food puffs.

^5^ Includes commercial and homemade pureed baby-food fruit and non-baby-food fruit; excludes 100% juice.

^6^ Includes any 100% fruit juice regardless of whether it is specifically labeled for babies or not. Beverages that are <100% fruit juice are included in sugar sweetened beverages.

^7^ Includes dark green, orange, red, starchy, and other vegetables, whether baby food or not, as well as white potatoes.

^8^ Includes commercial and homemade pureed baby-food vegetables.

^9^ Includes non-baby-food dark green, orange, red, starchy, and other vegetables; excludes baby food and white potatoes.

^10^ Includes fried potatoes, mashed potatoes and mixtures, and baked potatoes.

^11^ Includes meats and non-meat sources of protein, including cheese and yogurt.

^12^ Includes dried beans and legumes; eggs; vegetarian meat substitutes; nuts, nut butters, and seeds; cheese; and yogurt.

^13^ Includes cakes, pies, chocolate/sweet cookies, bars, brownies, sweet rolls, doughnuts, muffins, quick breads; frozen desserts, syrups, and sugar; as well as sugar sweetened beverages.

^14^ Includes chips, corn chips, popcorn, snack mix, and puffs (non-babyfood).

Table 5. Consumption of 100% juice in excess of 2017 AAP recommended amount of 4 oz (118 mL) or 2001 AAP recommended amount of 4-6 oz (118-177 mL) per day, children ages 12 to 23.9 mo^1^

| Amount consumed | Child’s age, mo | | | |
| --- | --- | --- | --- | --- |
|  | 12-14.9  (*n*=412) | 15-17.9  (*n*=308) | 18-20.9  (*n*=251) | 21-23.9  (*n*=162) |
| >4 oz [>118 mL] | 23±2.9 | 31±3.6 | 40±4.0 | 46±4.7 |
| >6 oz [>177 mL] | 14±2.2 | 23±3.3 | 29±3.8 | 32±4.6 |

^1^ Values are mean percentage of children consuming more than the 2017 AAP recommended 4 oz (118 mL) of juice per day ([39](#_ENREF_39)) or the 2001 AAP recommendation ([40](#_ENREF_40)) (reaffirmed in 2006 ([41](#_ENREF_41)) and in place at the time of the survey) of 4–6 oz (118–177 mL) per day, during a single 24-h recall ± SE.

Table 6. Consumption of selected foods during a single 24-h dietary recall, by race/ethnicity, children 0-23.9 mo

| Food Group | Consumers, %^1^ | | | Comparison Group^2^ | OR (95% CI)^3^ |
| --- | --- | --- | --- | --- | --- |
|  | Hisp. | NHW | NHB |  |  |
| 0-5.9 mo | | | | | |
|  | *n*=96 | *n*=389 | *n*=76 |  |  |
| Breastmilk | 59 | 53 | 43 | NHB | 0.63 (0.36, 1.12) |
| 6-11.9 mo | | | | | |
|  | *n*=125 | *n*=612 | *n*=113 |  |  |
| Breastmilk | 36 | 42 | 22 | NHB | 0.43 (0.26, 0.71) |
| RTE cereal^4^ | 16 | 13 | 5.3 | NHB | 0.33 (0.14, 0.77) |
| Baby-food puffs | 27 | 27 | 14 | NHB | 0.43 (0.24, 0.77) |
| 12-23.9 mo | | | | | |
|  | *n*=161 | *n*=770 | *n*=150 |  |  |
| Breastmilk | 11 | 13 | 5 | NHB | 0.36 (0.18, 0.72) |
| Sugar sweetened beverages | 28 | 25 | 45 | NHB | 2.31 (1.50, 3.57) |
| White potatoes^5^ | 28 | 30 | 46 | NHB | 2.03 (1.25, 3.30) |
| Cheese | 34 | 43 | 27 | NHB | 0.55 (0.35, 0.89) |
| Rice and pasta | 35 | 20 | 25 | Hispanic | 1.88 (1.06, 3.32) |
| Dried beans, peas, legumes | 14 | 9 | 8 | Hispanic | 1.84 (1.06, 3.20) |
| Eggs and egg dishes | 30 | 21 | 24 | Hispanic | 1.74 (1.14, 2.64) |
| Sweet bakery^6^ | 22 | 36 | 37 | Hispanic | 0.51 (0.31, 0.82) |
| Any fruit^7^ | 70 | 78 | 63 | NHW | 1.72 (1.20, 2.48) |
| Orange & red vegetables | 20 | 26 | 15 | NHW | 1.59 (1.08, 2.32) |
| Any 100% juice^8^ | 55 | 37 | 56 | NHW | 0.48 (0.35, 0.67) |

^1^ Values are mean percentage of children consuming the food category during a single 24-h recall.

^2^ The comparison group was the group with the highest or lowest percent consuming among the three groups that was also the most different from the value of the middle group. For example, for dried beans, peas, legumes, the group with the highest percent consuming is Hispanic and the lowest is NHB; we chose Hispanic for the comparison group because the middle value (9%, NHW) is much closer to the value for NHB (8%) than the value for Hispanic (14%).

^3^ Values are odds ratio for the percent consuming in the comparison group compared to the percent consuming for the other two groups, and the 95% confidence interval around the odds ratio.

^4^ Includes any ready-to-eat cereal that is not infant cereal.

^5^ Includes fried potatoes, mashed potatoes and mixtures, and baked potatoes.

^6^ Includes cakes, pies, chocolate/sweet cookies, bars, brownies, sweet rolls, doughnuts, muffins, and quick breads.

^7^ Includes any fruit whether baby food or not’ excludes 100% fruit juice.

^8^ Includes any 100% fruit juice regardless of whether it is specifically labeled for babies or not. Beverages that are <100% fruit juice are included in sugar sweetened beverages.

1. Online Supporting Materials: 4 tables [↑](#footnote-ref-2)
2. Abbreviations: AAP—American Academy of Pediatrics; FITS—Feeding Infants and Toddlers Study; NDSR—Nutrient Data System for Research; NHB—non-Hispanic black; NHW—non-Hispanic white; SSB—sugar-sweetened beverage; WIC—Special Supplemental Nutrition Program for Women, Infants, and Children. [↑](#footnote-ref-3)
3. Financial Support: Nestlé Research Center, Vers-Chez-les-Blanc, Route du Jorat 57, Case Postale 44, 1000 Lausanne-26, Switzerland [↑](#footnote-ref-4)
4. Conflict of Interest and Funding Disclosure: Roess, Catellier, Lutes, Anater—no conflicts of interest; Dietz—Weight Watchers Scientific Advisory Committee, grant support from Bridgespan for analyses of NHANES; Jacquier—employee of the Nestlé Research Center (funding source); Carvalho—employee of Nestlé Nutrition. [↑](#footnote-ref-5)
