# Supplementary material for: Food Consumption Patterns of Infants and Toddlers: Findings from the Feeding Infants and Toddlers Study (FITS) 2016
Source: J Nutr. 2018 Aug 31;148(Suppl 3):1525S–1535S. doi: 10.1093/jn/nxy171 (PMC6126630; doi:10.1093/jn/nxy171)
Supplement: Supplement Files [file nxy171_supplement_files.zip › nut264945-file002.docx]

# SupplementaRY DATA

**Supplemental Table 1. Demographic characteristics of the FITS2016 sample for ages 0 to 23.9 mo (unweighted) compared to 2014 US population^1^**

| **Characteristic** | **FITS 2016 Sample** | | | | **2014 US Population^2^** |
| --- | --- | --- | --- | --- | --- |
|  | **0-23.9 mo** | **0-5.9 mo** | **6-11.9 mo** | **12-23.9 mo** |  |
| Sample size (*n*) | 2,635 | 600 | 902 | 1,133 |  |
| Child’s race/ethnicity | | | | | |
| Hispanic | 15±0.6 | 16±1.50 | 14±1.16 | 14±1.04 | 26 |
| Non-Hispanic white | 67±0.8 | 65±1.95 | 68±1.55 | 68±1.38 | 50 |
| Non-Hispanic black | 13±0.6 | 13±1.36 | 13±1.11 | 13±1.00 | 13 |
| Other non-Hispanic | 5.0±0.4 | 6.3±1.00 | 5±0.73 | 4.3±0.61 | 11 |
| Primary caregiver’s education | | | | | |
| < High school | 3.9±0.4 | 4.7±0.86 | 3.9±0.64 | 3.4±0.54 | 13 |
| High school | 18±0.7 | 19±1.60 | 18±1.29 | 18±1.15 | 22 |
| Some college/post-secondary | 24±0.7 | 27±1.80 | 24±1.43 | 23±1.25 | 30 |
| Completed college | 39±0.9 | 37±1.97 | 38±1.62 | 41±1.46 | 22 |
| Some graduate work/degree | 14±0.6 | 13±1.36 | 15±1.19 | 15±1.05 | 14 |
| Household income | | | | | |
| Less than $10,000 | 8.4±0.5 | 9.3±1.19 | 8±0.90 | 8.2±0.81 | 6.8 |
| $10,000 to $19,999 | 8.7±0.5 | 9.3±1.19 | 9.2±0.96 | 8.1±0.81 | 8.6 |
| $20,000 to $34,999 | 19±0.7 | 18±1.57 | 21±1.35 | 18±1.13 | 14 |
| $35,000 to $49,999 | 17±0.7 | 17±1.54 | 18±1.29 | 17±1.11 | 13 |
| $50,000 to $74,999 | 20±0.7 | 20±1.64 | 19±1.30 | 22±1.23 | 18 |
| $75,000 to $99,999 | 13±0.6 | 14±1.39 | 13±1.14 | 13±1.00 | 13 |
| $100,000 to $149,999 | 10±0.5 | 8.3±1.13 | 8.9±0.95 | 11±0.91 | 15 |
| $150,000 or more | 3.9±0.34 | 3.7±0.77 | 2.9±0.56 | 4.1±0.59 | 11 |
| Other | | | | | |
| Respondent married | 71±0.8 | 71±1.86 | 72±1.50 | 71±1.35 | 81 |
| Mother employed | 44±1.0 | 37±2.17 | 43±1.82 | 49±1.66 | NA |
| Household receives SNAP | 25±0.8 | 24±1.75 | 26±1.45 | 26±1.28 | NA |
| Child ever breastfed | 83±0.7 | 86±1.41 | 85±1.20 | 81±1.17 | NA |

^1^ Values are percentage of respondents or US population ± SE; SE not available for US population. SNAP = Supplemental Nutrition Assistance Program

^2^ Percentage of population in US households in 2014 with at least one child under 4 y.

**Supplemental Table 2. Consumption of selected foods during a single 24-h dietary recall by 3-mo age intervals, children ages 6 to 23.9 mo^1^**

| **Food Group** | **Child's Age, mo** | | | | | |
| --- | --- | --- | --- | --- | --- | --- |
|  | **6-8.9**  **(n=468)** | **9-11.9**  **(n=434)** | **12-14.9**  **(n=412)** | **15-17.9**  **(n=308)** | **18-20.9**  **(n=251)** | **21-23.9**  **(n=162)** |
| Any grains | 77 | 91 | 96 | 93 | 96 | 97 |
| Infant cereal^2^ | 55 | 49 | 23 | 8.5 | 5.3 | 5.2 |
| Any fruit^3^ | 70 | 79 | 79 | 71 | 78 | 81 |
| Any 100% juice^4^ | 22 | 33 | 45 | 45 | 52 | 58 |
| Any vegetable^5^ | 76 | 68 | 73 | 74 | 71 | 71 |
| Baby-food vegetables | 51 | 39 | 11 | 6.0 | 3.5 | 1.5 |
| Non-baby-food vegetables^6^ | 23 | 34 | 56 | 60 | 58 | 62 |
| White potatoes | 12 | 18 | 34 | 31 | 33 | 30 |
| Meats and other proteins^7^  (excluding cheese and yogurt) | 24 | 52 | 76 | 85 | 87 | 84 |
| Baby-food meats | 5.0 | 3.4 | 1.5 | 0.3 | 1.3 | 0 |
| Meats (not baby food)^8^ | 12 | 40 | 62 | 73 | 73 | 72 |
| Beef | 1.3 | 3.6 | 11 | 12 | 11 | 13 |
| Chicken or turkey | 8.6 | 25 | 38 | 39 | 45 | 39 |
| Hotdogs, cold cuts, sausages, bacon | 2.4 | 11 | 21 | 31 | 28 | 27 |
| Other (non-meat) protein sources | 11 | 22 | 43 | 48 | 49 | 44 |
| Any sweets | 23 | 44 | 71 | 76 | 79 | 81 |
| Sugar sweetened beverages | 3.5 | 14 | 28 | 27 | 33 | 29 |
| Savory snacks | 2.8 | 8.5 | 13 | 20 | 18 | 22 |

^1^ Values are mean percentage of children consuming the food category during a single 24-h recall

^2^ Includes any kind of baby-food cereal, regardless of grain (i.e., rice, oat, quinoa, wheat, multigrain, or unknown grain)

^3^ Includes both baby-food fruit and non-baby-food fruit; excludes 100% juice.

^4^ Includes both baby 100% juice and regular 100% juice.

^5^ Includes any vegetable, including white potatoes.

^6^ Excludes white potatoes.

^7^ Excludes cheese and yogurt.

^8^ In addition to the categories listed, includes fish and shellfish, pork/ham, lamb, goat, game, and organ meats.

**Supplemental Table 3. Consumption of foods during a single 24-h dietary recall by race/ethnicity, children ages 0 to 11.9 mo^1^**

|  | **Child's Age, mo** | | | | | | | | |
| --- | --- | --- | --- | --- | --- | --- | --- | --- | --- |
| **Food Group** | **0-5.9** | | | |  | **6-11.9** | | | |
|  | **Overall**  **(n=600)** | **Hisp.**  **(n=96)** | **NHW**  **(n=389)** | **NHB**  **(n=76)** |  | **Overall**  **(n=902)** | **Hisp.**  **(n=126)** | **NHW**  **(n=617)** | **NHB**  **(n=114)** |
| Baby milks^2^ | 99 | 98 | 100 | 94 |  | 94 | 95 | 95 | 95 |
| Breastmilk | 54 | 59 | 53 | 43 |  | 39 | 36 | 42 | 22 |
| Infant formula | 62 | 63 | 59 | 72 |  | 65 | 68 | 61 | 81 |
| Non-baby milks^3^ | 1.9 | 1.6 | 0.2 | 7.4 |  | 11 | 8.6 | 10 | 7.6 |
| Any cow's milk^4^ | 1.9 | 1.6 | 0.2 | 7.4 |  | 10 | 7.8 | 9.4 | 7.6 |
| Whole milk | 1.4 | 1.6 | 0.2 | 4.5 |  | 5.8 | 2.6 | 5.3 | 5.1 |
| Reduced fat (2%) milk | 0.2 | 0 | 0 | 0.6l |  | 3.5 | 4.6 | 3.4 | 1.6 |
| Lowfat (1%) milk | 0.2 | 0 | 0 | 1.9 |  | 0.9 | 0.7 | 0.9 | 0.9 |
| Nonfat milk | 0.1 | 0 | 0 | 0.4 |  | 0.1 | 0% | 0.2 | 0 |
| Plant milks/dairy substitutes^5^ | 0 | 0 | 0 | 0 |  | 0.5 | 0.3 | 0.7 | 0 |
| Any flavored milk^6^ | 0 | 0 | 0 | 0 |  | 0.3 | 0.3 | 0.5 | 0 |
| Any grain products | 27 | 28 | 28 | 39 |  | 84 | 83 | 83 | 84 |
| Infant cereal^7^ | 25 | 24 | 26 | 37 |  | 52 | 45 | 52 | 56 |
| Family cereal^8^ | 0.7 | 1.6 | 0.5 | 0.6 |  | 20 | 23 | 18 | 22 |
| Presweetened | 0.3 | 1 | 0 | 0 |  | 5.1 | 8.8 | 3.1 | 8.9 |
| Not presweetened | 0.5 | 0.6 | 0.5 | 0.6 |  | 15 | 15 | 15 | 14 |
| Breads, rolls, biscuits, bagels, tortillas | 0.8 | 0.8 | 0.1 | 4.6 |  | 12 | 11 | 13 | 17 |
| Crackers, pretzels, rice cakes | 0.1 | 0 | 0 | 0.6 |  | 7.0 | 5.0 | 7.8 | 6.2 |
| Pancakes, waffles, French toast | 0.2 | 0 | 0 | 1.8 |  | 4.1 | 5.7 | 4.1 | 1.8 |
| Rice and pasta | 1.0 | 3.5 | 0.1 | 0 |  | 13 | 13 | 9.5 | 11 |
| Baby finger foods | 2.2 | 1.8 | 1.8 | 3.7 |  | 33 | 34 | 34 | 24 |
| Puffs | 1.4 | 0.5 | 1.2 | 2.0 |  | 25 | 27 | 27 | 14 |
| Any fruit^9^ | 17 | 20 | 14 | 22 |  | 74 | 68 | 78 | 82 |
| Baby-food fruit | 13 | 16 | 11 | 15 |  | 49 | 46 | 53 | 55 |
| Fruit (not baby food) | 4.9 | 6.2 | 3.5 | 7.3 |  | 36 | 30 | 40 | 34 |
| Fresh or frozen fruit | 3.7 | 5.5 | 2.3 | 5.0 |  | 31 | 28 | 34 | 27 |
| Canned or cooked fruit | 2.0 | 3.0 | 1.6 | 2.3 |  | 10 | 5 | 12 | 12 |
| Sweetened/syrup pack^10^ | 0.3 | 0.5 | 0.5 | 0 |  | 2.7 | 1.1 | 3.3 | 3.8 |
| Unsweetened/juice or water pack^10^ | 1.5 | 1.8 | 1.3 | 2.3 |  | 7.0 | 3.5 | 8.6 | 8.1 |
| Dried fruit | 0 | 0 | 0 | 0 |  | 1.1 | 0 | 1.5 | 1.4 |
| Any 100% juice^11^ | 4.6 | 4.0 | 4.0 | 9.0 |  | 27 | 34 | 21 | 41 |
| Baby 100% juice | 2.1 | 0.3 | 3.1 | 3.8 |  | 11 | 15 | 7.0^.^ | 18 |
| 100% Juice (not baby) | 2.7 | 3.7 | 1.2 | 6.8 |  | 17 | 19 | 14 | 24 |
| Any vegetable^12^ | 13 | 10 | 13 | 19 |  | 72 | 71 | 71 | 71 |
| Baby-food vegetables | 10 | 8.2 | 11 | 12 |  | 45 | 50 | 44 | 42 |
| Dark green baby-food vegetables^13^ | 0.2 | 0 | 0.3 | 0.6 |  | 1.6 | 0.7 | 2.5 | 0.9 |
| Orange & red baby-food vegetables^14^ | 7.3 | 5.9 | 7.5 | 9.4 |  | 31 | 36 | 28 | 29 |
| Starchy baby-food vegetables^15^ | 0.8 | 0 | 1.3 | 1.2 |  | 7.8 | 7.0 | 7.7 | 12 |
| Other baby-food vegetables^16^ | 2.5 | 2.3 | 3.0 | 2.7 |  | 15 | 15 | 16 | 16 |
| Vegetables (not baby food)^12^ | 3.1 | 3.2 | 1.9 | 0.7 |  | 29 | 23 | 31 | 24 |
| Dark green vegetables^17^ | 0.4 | 0 | 0.2 | 0 |  | 5.0 | 3.0 | 4.5 | 8.1 |
| Orange & red vegetables^18^ | 1.6 | 0.9 | 1.2 | 0 |  | 17 | 15 | 18 | 11 |
| White potatoes^19^ | 1.3 | 0.5 | 0.9 | 5.9 |  | 15 | 17 | 11 | 26 |
| French fries/other fried | 0.0 | 0 | 0 | 0 |  | 4.4 | 5.8 | 2.8 | 7.7 |
| Mashed potatoes | 1.1 | 0 | 0.7 | 5.9 |  | 8.4 | 8.4 | 6.0 | 20 |
| Other starchy vegetables^20^ | 0.3 | 0 | 0.5 | 0.7 |  | 5.1 | 3.3 | 6.7 | 5.0 |
| Other vegetables^21^ | 1.4 | 2.3 | 0.4 | 0.7 |  | 12 | 9.2 | 12 | 8.2 |
| Any meat or other protein food | 3.1 | 3.5 | 1.2 | 6.4 |  | 41 | 42 | 39 | 47 |
| Baby-food meats | 0.5 | 0 | 0.7 | 1 |  | 4.2 | 4.5 | 3.8 | 6.2 |
| Meats (not baby food)^22^ | 1.2 | 1.8 | 0 | 5.3 |  | 26 | 24 | 25 | 29 |
| Beef | 0.1 | 0.2 | 0 | 0 |  | 2.5 | 1.0 | 3.1 | 4.1 |
| Chicken or turkey | 1.0 | 1.6 | 0 | 4.6 |  | 17 | 19 | 13 | 22 |
| Fish and shellfish | 0 | 0 | 0 | 0 |  | 1.6 | 1.2 | 1.6 | 2.8 |
| Hotdogs, cold cuts, sausages, bacon | 0.9 | 1.6 | 0 | 3.6 |  | 7.0 | 1.3 | 9.3 | 10 |
| Pork/ham | 0 | 0 | 0 | 0 |  | 1.6 | 1.9 | 1.4 | 0 |
| Other protein sources | 2.5 | 3.5 | 0.5 | 4.3 |  | 26 | 26 | 27 | 27 |
| Dried beans, peas, legumes | 0.8 | 1.9 | 0.1 | 0 |  | 3.9 | 3.4 | 4.4 | 1.5 |
| Vegetarian meat substitutes | 0.0 | 0 | 0 | 0 |  | 0.5 | 1.3 | 0.4 | 0 |
| Eggs and egg dishes | 0.8 | 1.7 | 0.2 | 0.7 |  | 11 | 16 | 10 | 15 |
| Nuts, nut butters, and seeds | 0.0 | 0 | 0 | 0 |  | 2.4 | 2.0 | 2.7 | 1.9 |
| Peanut butter | 0.0 | 0 | 0 | 0 |  | 2.0 | 1.5 | 2.6 | 1.9 |
| Cheese | 1.0 | 1.4 | 0 | 3.6 |  | 9.0 | 4.6 | 11 | 13 |
| Yogurt | 0.8 | 1.4 | 0.3 | 0 |  | 7.5 | 6.5 | 9.6 | 2.1 |
| Any sweets or sugar-sweetened beverage | 3.1 | 2.8 | 3.1 | 3.5 |  | 34 | 38 | 32 | 36 |
| Cereal/nutrition bars | 0.0 | 0 | 0.1 | 0 |  | 1.0 | 0 | 0.8 | 0.7 |
| Sweet bakery^23^ | 0.6 | 1.0 | 0 | 1.3 |  | 8.0 | 7.4 | 8.5 | 8.6 |
| Candy | 0.3 | 0.5 | 0.2 | 0.7 |  | 1.2 | 1.4 | 1.1 | 2.1 |
| Ice cream, frozen yogurt, pudding | 0.1 | 0 | 0 | 0.8 |  | 2.8 | 4.9 | 2.6 | 1.4 |
| Gelatins, ices, sorbets | 0.0 | 0 | 0 | 0 |  | 0.8 | 0.4 | 0.8 | 2.4 |
| Milk flavorings | 0.0 | 0 | 0 | 0 |  | 0.1 | 0 | 0.2 | 0 |
| Sugar, syrups, preserves, jelly | 0.6 | 0 | 0.8 | 1.7 |  | 4.6 | 5.2 | 4.3 | 5.1 |
| Baby-food desserts | 0.7 | 0.7 | 0.9 | 0.4 |  | 8.1 | 7.8 | 8.4 | 9.0 |
| Baby-food cookies, teething biscuits | 0.8 | 1.3 | 0.2 | 0 |  | 9.9 | 12 | 9.0 | 13 |
| Sugar sweetened beverages | 0.9 | 0.5 | 0.9 | 1.3 |  | 8.5 | 15 | 6.4 | 7.3 |
| Soft drinks | 0.0 | 0 | 0 | 0 |  | 0.6 | 0.7 | 0.3 | 2.4 |
| Fruit flavored drinks | 0.8 | 0.5 | 0.9 | 0.6 |  | 7.3 | 14 | 4.6 | 6.6 |
| Tea and coffee | 0.2 | 0.5 | 0 | 0.8 |  | 0.5 | 0 | 0.9 | 0 |
| Sports drinks | 0.0 | 0 | 0 | 0 |  | 0.5 | 0 | 0.2 | 3.0 |
| Savory snacks^24^ | 0.5 | 1.6 | 0 | 0.7 |  | 5.7 | 9.2 | 3.1 | 5.6 |

^1^ Values are mean percentage of children consuming the food category during a single 24-h recall; NH=non-Hispanic; NHW=non-Hispanic white; NHB=non-Hispanic black. Note that n for Hispanic, NHW, and NHB sum to less than n overall because the overall value includes non-Hispanic other race, which is not shown due to small cell size.

^2^ Includes human milk (breastmilk), infant formula, and toddler milk drinks

^3^ Includes cow’s milk, goat’s milk, plant milks, and other milk substitutes

^4^ Includes all fat levels, as well as flavored, unflavored, or powdered.

^5^ Includes soy milk, almond milk, and other plant-based dairy substitutes; may be flavored or unflavored.

^6^ Includes flavored cow’s milk, flavored plant milks, and flavored dairy substitutes.

^7^ Includes any kind of baby-food cereal, regardless of grain (i.e., rice, oat, quinoa, wheat, multigrain, or unknown grain)

^8^ Includes any ready-to-eat or hot cereal; excludes infant cereals.

^9^ Includes both baby-food fruit and non-baby-food fruit; excludes 100% juice.

^10^ Sweetened and unsweetened apply only to canned or cooked fruit

^11^ Includes both baby 100% juice and regular 100% juice.

^12^ Includes any vegetable, including white potatoes.

^13^ Includes baby-food broccoli, spinach, and broccoli or spinach mixtures.

^14^ Includes baby-food beets; carrots; squash; sweet potato; and carrot, squash, or sweet potato mixtures.

^15^ Includes baby-food corn, green peas, and other starchy vegetables other than white potatoes, as well as corn and pea mixtures.

^16^ Includes baby-food green beans, green bean mixtures, and other baby-food vegetable mixtures not included elsewhere.

^17^ Includes broccoli, Brussel sprouts, greens, and spinach; excludes baby food.

^18^ Includes beets, carrots, squash, sweet potato, red peppers, tomatoes, and tomato sauce; excludes baby food.

^19^ Includes French fries, any other kind of fried potatoes, mashed potatoes and mixtures, and baked potatoes.

^20^ Includes corn, green peas, and other starchy vegetables other than white potatoes; excludes baby food.

^21^ Includes asparagus, cabbage, cauliflower, celery, cucumber, green beans, lettuce, green salad, mushrooms, onions, pea pods, peppers (not red), zucchini/summer squash, and vegetable mixtures; excludes baby food.

^22^ In addition to the categories listed, includes lamb, goat, game, and organ meats, which are consumed by less than 1% of respondents in all age groups.

^23^ Includes cakes, pies, chocolate/sweet cookies, bars, brownies, sweet rolls, doughnuts, muffins, and quick breads.

^24^ Includes chips, corn chips, popcorn, snack mix, and puffs (non-babyfood).

**Supplemental Table 4. Consumption of foods during a single 24-h dietary recall by race/ethnicity, children ages 12 to 23.9^1^**

|  | **Child's Age, mo** | | | | | | | | |
| --- | --- | --- | --- | --- | --- | --- | --- | --- | --- |
| **Food Group** | **12-17.9** | | | |  | **18-23.9** | | | |
|  | **Overall**  **(n=720)** | **Hisp.**  **(n=104)** | **NHW**  **(n=499)** | **NHB**  **(n=81)** |  | **Overall**  **(n=413)** | **Hisp.**  **(n=57)** | **NHW**  **(n=273)** | **NHB**  **(n=69)** |
| Baby milks^2^ | 26 | 19 | 31 | 16 |  | 6.3 | 9.1 | 5.6 | 2.4 |
| Breastmilk | 18 | 13 | 22 | 11 |  | 5.1 | 9.1 | 3.6 | 0.8 |
| Infant formula | 7.3 | 6.8 | 7.5 | 3.9 |  | 1.1 | 0 | 1.6 | 1.6 |
| Non-baby milks^3^ | 86 | 91 | 83 | 87 |  | 88 | 91 | 86 | 93 |
| Any cow's milk^4^ | 81 | 88 | 81 | 80 |  | 85 | 87 | 81 | 90 |
| Whole milk | 69 | 75 | 67 | 68 |  | 65 | 72 | 59 | 66 |
| Reduced fat (2%) milk | 11 | 8.3 | 13 | 8.5 |  | 18 | 16 | 19 | 17 |
| Lowfat (1%) milk | 3.2 | 4.4 | 2.6 | 4.8 |  | 6.2 | 4.9 | 6.6 | 10 |
| Nonfat milk | 2.1 | 3.5 | 2.4 | 0 |  | 3.0 | 1.2 | 2.4 | 5.7 |
| Plant milks/dairy substitutes^5^ | 5.1 | 2.4 | 3.7 | 7.1 |  | 5.1 | 3.8 | 7 | 3.9 |
| Any flavored milk^6^ | 4.3 | 4.6 | 3.7 | 3.3 |  | 8 | 5.5 | 11 | 7 |
| Any grain products | 94 | 93 | 95 | 96 |  | 96 | 98 | 95 | 97 |
| Infant cereal^7^ | 16 | 9.1 | 14 | 21 |  | 5.3 | 7.1 | 4.2 | 5.4 |
| Family cereal^8^ | 50 | 44 | 50 | 54 |  | 58 | 55 | 54 | 71 |
| Presweetened | 23 | 14 | 24 | 27 |  | 30 | 29 | 29 | 41 |
| Not presweetened | 30 | 31 | 30 | 29 |  | 31 | 27 | 28 | 35 |
| Breads, rolls, biscuits, bagels, tortillas | 39 | 44 | 44 | 33 |  | 47 | 51 | 50 | 40 |
| Crackers, pretzels, rice cakes | 31 | 26 | 38 | 19 |  | 41 | 44 | 42 | 40 |
| Pancakes, waffles, French toast | 16 | 14 | 19 | 14 |  | 18 | 11 | 21 | 19 |
| Rice and pasta | 28 | 33 | 19 | 24 |  | 26 | 37 | 20 | 25 |
| Baby finger foods | 16 | 19 | 18 | 8.8 |  | 5.7 | 8.4 | 6.0 | 2.8 |
| Puffs | 11 | 13 | 11 | 5.4 |  | 3.4 | 4.6 | 3.8 | 1.7 |
| Any fruit^9^ | 75 | 78 | 80 | 68 |  | 79 | 78 | 80 | 68 |
| Baby-food fruit | 13 | 3.5 | 7.4 | 2.4 |  | 6.2 | 3.5 | 7.4 | 2.4 |
| Fruit (not baby food) | 70 | 77 | 79 | 68 |  | 78 | 77 | 79 | 68 |
| Fresh or frozen fruit | 59 | 73 | 70 | 63 |  | 70 | 73 | 70 | 63 |
| Canned or cooked fruit | 22 | 10 | 28 | 17 |  | 22 | 10 | 28 | 17 |
| Sweetened/syrup pack^10^ | 11 | 5.6 | 10 | 10 |  | 9.6 | 5.6 | 10 | 10 |
| Unsweetened/juice or water pack^10^ | 11 | 4.2 | 16 | 5.9 |  | 11 | 4.2 | 16 | 5.9 |
| Dried fruit | 3.8 | 1.6 | 12 | 3.4 |  | 8.6 | 1.6 | 12 | 3.4 |
| Any 100% juice^11^ | 45 | 61 | 45 | 72 |  | 55 | 61 | 45 | 72 |
| Baby 100% juice | 7.6 | 3.1 | 2.3 | 12 |  | 4.8 | 3.1 | 2.3 | 12 |
| 100% Juice (not baby) | 39 | 58 | 43 | 65 |  | 51 | 58 | 43 | 65 |
| Any vegetable^12^ | 73 | 72 | 72 | 76 |  | 71 | 63 | 73 | 76 |
| Baby-food vegetables | 8.7 | 12 | 7.6 | 9.2 |  | 2.5 | 4.1 | 2.6 | 0.8 |
| Any vegetable (non-baby-food) EXCEPT white potatoes^13^ | 58 | 47 | 61 | 50 |  | 60 | 52 | 63 | 61 |
| Dark green vegetables^14^ | 12 | 9.0 | 11 | 11 |  | 14 | 14 | 13 | 18 |
| Orange & red vegetables^15^ | 25 | 20 | 23 | 13 |  | 25 | 20 | 29 | 15 |
| Other starchy vegetables^16^ | 16 | 11 | 19 | 16 |  | 15 | 9.0 | 17 | 16 |
| Other vegetables^17^ | 2.5 | 4.3 | 2.1 | 3.4 |  | 0.2 | 0 | 0.5 | 0 |
| White potatoes^18^ | 33 | 44 | 28 | 43 |  | 32 | 29 | 26 | 42 |
| French fries/other fried | 11 | 17 | 10 | 13 |  | 15 | 13 | 11 | 20 |
| Mashed potatoes | 15 | 18 | 14 | 22 |  | 13 | 13 | 11 | 20 |
| Any meat or other protein food | 88 | 87 | 90 | 91 |  | 91 | 85 | 92 | 94 |
| Baby-food meats | 0.9 | 0 | 1.2 | 2.6 |  | 0.6 | 0 | 0.9 | 1 |
| Meats (not baby food)^19^ | 68 | 71 | 67 | 83 |  | 72 | 71 | 71 | 79 |
| Beef | 11 | 13 | 11 | 17 |  | 12 | 12 | 14 | 12 |
| Chicken or turkey | 39 | 38 | 38 | 46 |  | 42 | 49 | 37 | 42 |
| Fish and shellfish | 6.8 | 4.9 | 6.3 | 12 |  | 7 | 4.9 | 6.1 | 11 |
| Hotdogs, cold cuts, sausages, bacon | 26 | 26 | 26 | 31 |  | 27 | 24 | 28 | 34 |
| Pork/ham | 4.6 | 4.8 | 2.9 | 1.2 |  | 4 | 2.2 | 4.4 | 7.9 |
| Other protein sources | 68 | 73 | 72 | 61 |  | 75 | 74 | 76 | 73 |
| Dried beans, peas, legumes | 10 | 18 | 7.8 | 6.3 |  | 9.4 | 11 | 9.2 | 9.1 |
| Vegetarian meat substitutes | 1 | 0 | 0.8 | 2.8 |  | 1.6 | 3 | 1.1 | 1.6 |
| Eggs and egg dishes | 29 | 41 | 26 | 29 |  | 26 | 33 | 24 | 29 |
| Nuts, nut butters, and seeds | 14 | 13 | 15 | 11 |  | 20 | 16 | 25 | 8.4 |
| Peanut butter | 11 | 11 | 13 | 8.9 |  | 16 | 14 | 21 | 5.4 |
| Cheese | 32 | 33 | 41 | 20 |  | 40 | 35 | 45 | 32 |
| Yogurt | 22 | 20 | 24 | 14 |  | 25 | 15 | 28 | 26 |
| Any sweets or sugar-sweetened beverage | 73 | 75 | 70 | 77 |  | 80 | 76 | 81 | 78 |
| Cereal/nutrition bars | 4.8 | 5.6 | 6.8 | 0.9 |  | 8.3 | 10 | 11 | 2.0 |
| Sweet bakery^20^ | 27 | 19 | 29 | 28 |  | 36 | 26 | 42 | 43 |
| Candy | 8.4 | 10 | 10 | 6.6 |  | 17 | 18 | 18 | 15 |
| Ice cream, frozen yogurt, pudding | 6.4 | 6.3 | 5.3 | 6.3 |  | 8.1 | 8.2 | 7.7 | 6.6 |
| Gelatins, ices, sorbets | 2.7 | 3.3 | 2.2 | 1.2 |  | 4.9 | 7.3 | 4.2 | 5.6 |
| Milk flavorings | 1.1 | 0 | 1.0 | 0 |  | 1.3 | 1.3 | 1.8 | 0 |
| Sugar, syrups, preserves, jelly | 22 | 21 | 23 | 22 |  | 30 | 27 | 29 | 34 |
| Baby-food desserts | 6.8 | 12 | 5.9 | 0.8 |  | 5.1 | 12 | 3.1 | 2.6 |
| Baby-food cookies, teething biscuits | 11 | 13 | 10 | 13 |  | 8.5 | 8.2 | 7.0 | 4.8 |
| Sugar sweetened beverages | 27 | 29 | 19 | 42 |  | 31 | 27 | 31 | 47 |
| Soft drinks | 3.2 | 4.8 | 1.3 | 6 |  | 3.1 | 0 | 3.0 | 9.3 |
| Fruit flavored drinks | 21 | 23 | 15 | 35 |  | 26 | 23 | 25 | 42 |
| Tea and coffee | 3.3 | 3.2 | 2.6 | 1.8 |  | 1.4 | 1.1 | 1.7 | 0.9 |
| Sports drinks | 0.9 | 0 | 1.4 | 2.0 |  | 1.8 | 3.2 | 1.9 | 0 |
| Savory snacks^21^ | 17 | 15 | 19 | 28 |  | 20 | 13 | 23 | 29 |

^1^ Values are mean percentage of children consuming the food category during a single 24-h recall; NH=non-Hispanic; NHW=non-Hispanic white; NHB=non-Hispanic black. Note that n for Hispanic, NHW, and NHB sum to less than n overall because the overall value includes non-Hispanic other race, which is not shown due to small cell size.

^2^ Includes human milk (breastmilk), infant formula, and toddler milk drinks

^3^ Includes cow’s milk, goat’s milk, plant milks, and other milk substitutes

^4^ Includes all fat levels, as well as flavored, unflavored, or powdered.

^5^ Includes soy milk, almond milk, and other plant-based dairy substitutes; may be flavored or unflavored.

^6^ Includes flavored cow’s milk, flavored plant milks, and flavored dairy substitutes.

^7^ Includes any kind of baby-food cereal, regardless of grain (i.e., rice, oat, quinoa, wheat, multigrain, or unknown grain)

^8^ Includes any ready-to-eat or hot cereal; excludes infant cereals.

^9^ Includes both baby-food fruit and non-baby-food fruit; excludes 100% juice.

^10^ Sweetened and unsweetened apply only to canned or cooked fruit

^11^ Includes both baby 100% juice and regular 100% juice.

^12^ Includes any vegetable, including white potatoes and baby food.

^13^ Includes any non-baby-food vegetable, excludes white potatoes.

^14^ Dark green vegetables include broccoli, Brussel sprouts, greens, and spinach.

^15^ Red and orange vegetables include beets, carrots, squash, sweet potato, red peppers, tomatoes, and tomato sauce.

^16^ Starchy vegetables include corn, green peas, and other starchy vegetables other than white potatoes.

^17^ Other vegetables include asparagus, cabbage, cauliflower, celery, cucumber, green beans, lettuce, green salad, mushrooms, onions, pea pods, peppers (not red), zucchini/summer squash, and vegetable mixtures;

^18^ Includes French fries, any other kind of fried potatoes, mashed potatoes and mixtures, and baked potatoes.

^19^ In addition to the categories listed, includes lamb, goat, game, and organ meats, which are consumed by less than 1±% of respondents in all age groups.

^20^ Includes cakes, pies, chocolate/sweet cookies, bars, brownies, sweet rolls, doughnuts, muffins, and quick breads.

^21^ Includes chips, corn chips, popcorn, snack mix, and puffs (non-babyfood).
